# AQbD based green UPLC method to determine mycophenolate mofetil impurities and Identification of degradation products by QToF LCMS

**DOI:** 10.1038/s41598-022-22998-0

**Published:** 2022-11-09

**Authors:** Siva Krishna Muchakayala, Naresh Kumar Katari, Kalyan Kumar Saripella, Henele Schaaf, Vishnu Murthy Marisetti, Leela Prasad Kowtharapu, Sreekantha Babu Jonnalagadda

**Affiliations:** 1 Douglas Pharma US Inc, 1035 Louis Drive, Warminster, PA 18974 USA; 2 Department of Chemistry, GITAM School of Science, GITAM Deemed to be University, Hyderabad, Telangana 502329 India; 3 Analytical Research and Development, ScieGen Pharmaceuticals Inc, 89 Arkay Drive, Hauppauge, NY 11788 USA; 4 School of Chemistry & Physics, College of Agriculture, Engineering & Science, Westville Campus, University of KwaZulu-Natal, P Bag X 54001, Durban, 4000 South Africa

**Keywords:** Analytical chemistry, Mass spectrometry, Green chemistry

## Abstract

We report an ideal method for quantifying impurities in mycophenolate mofetil drug substances and their oral suspension preparations. We developed a systematic and eco-friendly analytical approach utilizing quality by design (QbD) and green chemistry principles. Initially, the critical method parameters (CMPs) were screened using a D-optimal design. The robust final method conditions were optimized using a systematic central composite design (CCD). Through graphical and numerical optimization, the protocol conditions were augmented. The pH of mobile phase buffer (25 mM KH_2_PO_4_) (MP-A), initial gradient composition (% MP-A), flow rate (mL min^−1^), and column oven temperatures (°C) are 4.05, 87, 0.4, and 30, respectively. The best possible separation between the critical pairs was achieved while using the Waters Acquity UPLC BEH C_18_ (100 × 2.1) mm, 1.7 µm analytical column. A mixture of water and acetonitrile in the ratio of 30:70 (v/v) was used as mobile phase-B for the gradient elution. The analytical method was validated in agreement with ICH and USP guidelines. The specificity results revealed that no peaks interfered with the impurities and MPM. The mean recovery of the impurities ranged between 96.2 and 102.7%, and the linearity results r > 0.999 across the range of LOQ – 150%. The precision results (%RSD) ranged between 0.8 and 4.5%. The degradation products formed during the base-induced degradation were identified as isomers of mycophenolic acid and sorbitol esters using Q-ToF LC–MS and their molecular and fragment ion peaks. The developed method eco-friendliness and greenness were assessed using analytical greenness (AGREE), green analytical procedure index (GAPI), and analytical eco score, and found it is green.

## Introduction

Mycophenolate mofetil (MPM) is an ester of Mycophenolic acid (MPA) and morpholine ethanol. It is a prodrug of MPA and appears as a white crystalline powder bearing the chemical name 2-Morpholinoethyl (E)-6-(4-hydroxy-6-methoxy-7-methyl-3-oxo-5-phthalanyl)-4-methyl-4-hexenoate, molecular formula C_23_H_31_NO_7_, and the molecular weight 433.49 Da^1^. It is insoluble in water and soluble in low pH buffer (1.2), acetone, and acetonitrile. MPM is an immunosuppressive agent mainly used along with cyclosporine or steroids during organ transplantation procedures, such as kidney, heart, and liver. A patient's autoimmune system senses a new organ and attempts to reject it during organ transplant surgery. The patient's autoimmune system must be suppressed to prevent the rejection of the transplanted organ. MPM is an inosine monophosphate dehydrogenase inhibitor that acts as an immuno-suppressing agent to avoid organ rejection.

The MPM rapidly hydrolyzes and forms the active metabolite mycophenolic acid (MPA). A portion of MPA is further metabolized and forms the glucuronide metabolite of MPA. Glucuronide metabolite releases MPA during hepatic recirculation. In the late 1970s, pure MPA was used as an immunosuppressive agent. However, it was discontinued due to gastrointestinal complications. Then, as an alternative, a rapidly hydrolyzed and easily absorbed prodrug of MPA called MPM is used to prevent organ rejection after transplantation.

The current research aims to develop a single analytical method for determining impurities in drug substances and products using QbD and green chemistry principles. An extensive literature survey found several impurity testing methods in the USP monograph for MPM drug substances and their formulated products, including capsules, injections, oral suspensions, and tablets^1^. The mobile and stationary phases described in the monograph methods are different; therefore, the procedures are not the same for the drug substances and formulations. All the processes consume higher volumes of mobile phase (1.5 mL min^−1^) with a runtime > 40 min. Few analytical methods are reported for determining impurities^2–4^, but these methods did not cover separating all the contaminants listed in Table 1. Further, various assay methods are reported for quantifying MPM from bulk and formulations using HPLC^5–11^, HPTLC^12^, and voltammetry techniques^13^. Analytical and bio-analytical methods for MPM and MPA simultaneous determination have been reported^14–28^. The reported assay and bio-analytical methods are intended to determine the active drug and its metabolite but not the impurities.

**Table 1:**
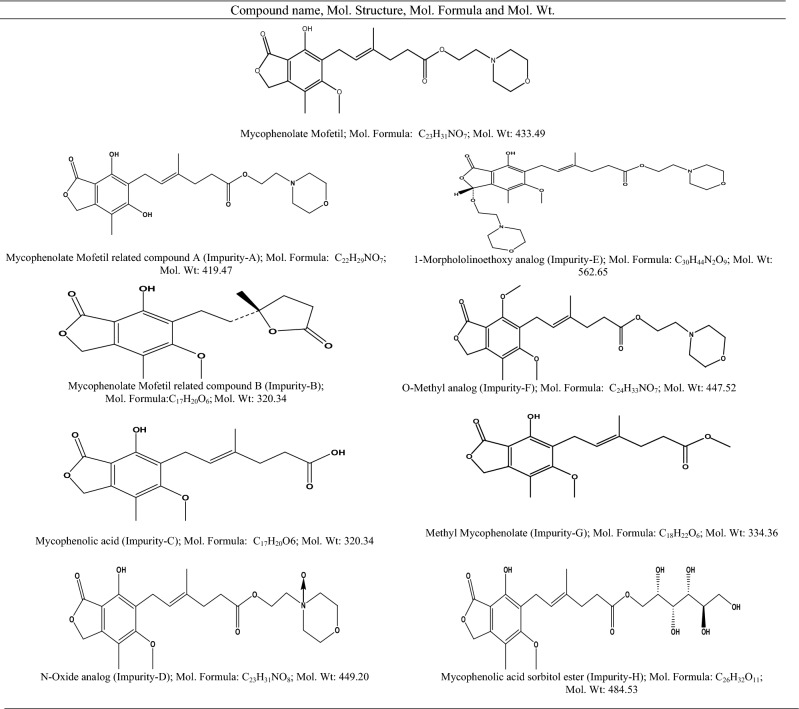
Chemical name, molecular structure, formula, and molecular weights of MPM and its impurities.

A traditional one-factor-at-a-time (OFAT) method development process is a time-consuming, trial and error approach^29–38^. The QbD-based method development process is a systematic approach that involves identifying probable risks that cause failures. Further steps include identifying critical method parameters (CMPs) followed by optimization using the design of experiments (DoE). Moreover, the QbD approach helps in understanding the magnitude of specific effects and the interaction effects of CMPs.

Due to the undetermined toxicological behavior of unknown impurities, the regulatory threshold limits are significantly lower than known impurities. Identifying the potential unknown contaminants generated during the stress studies is highly demanded, which helps to qualify the impurity through toxicology studies. This process enhances the drug product's safety, quality, and efficacy. The ultimate goal is to provide safer medicines for human and veterinary use. To identify the unknown degradation impurities, an LC–MS technique^39–44^ was utilized to determine the unknown impurities' molecular mass and fragmentation pattern.

Recently, green chemistry principles have been highly prioritized and used by most government and private industries. Green chemistry principles help to minimize environmental pollution such as water, air, and earth; and improve the better living life of humans and animals. "Green chemistry is the design of chemical products and processes that reduce or eliminate the use and generation of hazardous substances"^45,46^. The current research study aims to minimize the usage of organic solvents and other pollutants. Usually, analytical eco-scale, Analytical greenness (AGREE), national environmental method index (NEMI), and green analytical procedure index (GAPI) tools are used to determine the eco-friendly nature. The present research study focused on using AGREE, GAPI, and analytical eco-scale to assess the greenness of the method^47–53^.

Based on the literature review, this is the first-ever UPLC method constructed utilizing QbD and green chemistry principles to determine MPM impurities from its drug substances and oral suspension preparations. In addition, it is the primary method to identify the unknown impurities formed in a base stress study.

## Materials and methods

### Instrumentation and software

The Waters H-Class UPLC was utilized for the Method development and validation study (Waters Corporation, Milford, MA). The entire study was conducted using empower software to acquire, process, and report chromatographic data (Waters Corporation, Milford, MA). A statistical tool, Design-Expert-13, was employed to screen and optimize the CMPs (Stat-Ease Inc, Minneapolis, USA). Various Acquity UPLC columns (100 mm Length × 2.1 mm ID), such as BEH C_8_, BEH C_18_, BEH Phenyl, HSS T_3_, and Protein BEH C_4_ were assessed for the separation of components (Waters Corporation, Milford, MA). The Waters Xevo G2-XS Quadrupole time-of-flight (Q-ToF) MS instrument with step wave ion optics and XS collision cell coupled with Waters I-Class UPLC was used to separate and identify unknown impurities (Waters Corporation, Milford, MA). UNIFI software was used to identify molecular and fragment ions and their molecular structures (Waters Corporation, Milford, MA).

### Materials and reagents

The MPM, Impurity-A, and Impurity-B were obtained from USP (Rockville, Maryland, USA). The Impurity-H is supplied by Molcan Corporation (Toronto, ON, Canada). All other impurities (Impurity-C, D, E, F, and G) were obtained from Chunghwa Chemical Synthesis & Biotech CO., LTD (Shu-Lin, New Taipei, Taiwan). The mycophenolate mofetil oral suspension 200 mg mL^−1^ (Cellcept) was procured from Roche (Indianapolis, USA). The MPM and its impurity's chemical name and molecular details are summarized in Table 1. The mobile phase and sample solutions were prepared using high purity HPLC grade chemicals and solvents from EMD Millipore. Purified water from the Elga water system was used for the UPLC analysis. The chemicals, reagents, and solvents utilized for LCMS analysis were procured from Honeywell (Charlotte, NC, USA). The 0.2 µm syringe filters were bought from Whatman (Little Chalfont, Buckinghamshire, UK).

### Chromatographic conditions

The Waters Acquity UPLC BEH C_18_, (100 × 2.1) mm, 1.7 µm column separated the MPM, impurities, and peaks from the sample matrix. A 25 mM monobasic potassium phosphate buffer pH adjusted to 4.05 with orthophosphoric acid (OPA) solution was used as mobile phase-A. A mixture of water and ACN in the ratio of 30:70 (v/v) was used as mobile phase-B. A simple and linear binary gradient program (T(min)/%A/%B: 0.0–12.0/87 → 0/13 → 100) with a 0.4 mL min^−1^ was used to separate components. A three-minutes initial composition of mobile phases was acclimated to re-equilibrate the column before injecting any sample. The sample cooler and column oven temperatures were maintained at 10 °C and 30 °C, respectively. The 0.2 µm PVDF filters were used to filter the sample solutions. The chromatographic signal was monitored at 215 nm by injecting the 5.0 µL of sample solution.

### Preparation of diluent

The standard and impurity stock solutions were prepared in ACN. To achieve complete dissolution, a 0.1% formic acid in a mixture of water and ACN in the ratio of 80:20 (v/v) was used as a diluent to prepare a final standard and sample solution.

### Preparation of standard solutions

The 25 mg of MPM USPRS was accurately weighed, transferred into a 100 mL volumetric flask, and dissolved in ACN by sonication and mixing. A series of dilutions, such as 5.0 mL to 100 mL, was executed from the standard stock solution to attain the final concentration of 0.5 µg mL^−1^ of MPM (Standard solution).

### Preparation of sample solutions

Accurately weighed 1.2 g of oral suspension (Approximately 200 mg of MPM) in a clean 100 mL VF, added 60.0 mL of diluent, and closed firmly with a lid. The solution was vortex for 2 min, sonicated for 15 min, and diluted to volume with diluent. The 5.0 mL sample stock solution was diluted to 100 mL VF using a diluent and mixed well. Discarded the first 5 mL of filtrate from the filter (0.2 µm PVDF) and collected the sample in an HPLC vial for analysis.

### LC–MS method conditions for the identification of unknown impurities

To identify the unknown impurities generated during the base degradation, we used similar chromatographic conditions as the developed UPLC method by substituting non-volatile buffer and reagent solutions with volatile buffers and reagents. 25 mM ammonium acetate was used as a buffer, and glacial acetic acid (GAA) as a reagent. A mixture of 25 mM ammonium acetate (500 mL) and GAA (5.0 mL) (resultant solution pH ~ 3.8) was used as a mobile phase-A. A solution containing a mixture of water and ACN in the ratio of 30:70 was (v/v) used as mobile phase-B. A simple and linear-gradient program (T(min)/%A/%B: 0.0–5.0/85 → 70/15 → 30; 5.0–15.0/70 → 30/30 → 70) with 3 min re-equilibration was used to separate the components. The analytical column (Waters Acquity UPLC BEH C_18_, (100 × 2.1) mm, 1.7 µm), flow rate (0.4 mL min^−1^), sample cooler (10 °C), and column oven temperatures (30 °C) were considered from the developed UPLC method. Electrospray ionization (ESI) with positive and negative polarities was used in the study. The soft ionization was performed using the low collision energy (Low CE) (3.0 eV) to identify the molecular ion peak (M^+^ or M^−^). The hard ionization was performed on a collision energy ramp (High CE) ramp from 20.0 eV to 45.0 eV to identify the fragment ion peaks. Set cone voltage, source temperature, and desolvation temperature were 40 V, 100 °C, and 250 °C, respectively. Set cone and desolvation gas flows were 50 and 600 L/h, respectively. Leucine enkephalin solution (200 pg/µL) was used as a mass lock solution to correct the mass error.

## Results and discussion

The drug substance USP monograph method monitors most of the impurities of MPM, thus deciding to evaluate the feasibility of the current study. The feasibility study revealed that the analyte response was low, later peaks were eluted broadly due to band broadening, and longer analysis times make the USP monograph methods inapplicable. The obtained chromatogram from the feasibility study is shown in Supplementary Fig. 1.

### Analytical target profile (ATP), critical method parameters (CMPs), and critical method attributes (CMAs)

The quality target product profile (QTPP) is a set of comprehensive objectives of the drug product, such as purity, quality, efficacy, safety, and route of administration. Similarly, ATP is a set of analytical method objectives that outline the AQbD process. The prime focus of the current study is to develop an analytical method for accurately quantifying identified and unidentified impurities present in MPM and its oral suspension formulation with the shortest analysis time. Additionally, all the component peaks in the chromatogram shall be resolved with a minimum resolution (Rs > 1.5) between any critical peak pairs during the stress and stability studies.

The selection of CMAs and identification of CMPs is the most crucial phase in an AQbD process. Screening designs were used to identify CMPs and narrow them down for the final optimization method. The most probable CMPs for the current chromatographic method are buffer type and concentration, organic modifier, ion-pair reagents, flow rate, gradient program, and column oven temperature. The resolution between closely eluting peak pairs (Rs) and retention time (Rt) of the most retaining peak in the column were considered CMAs for the current study.

### Risk assessment

The risk assessment helps to identify the CMPs that have a substantial influence on CMAs and ATP. In QbD-based method development, the risks contributing to CMAs and ATP's failure must be studied extensively. The Ishikawa fishbone diagram is used to establish a cause-and-effect relationship between CMPs and ATP. The main risks identified for the current study are the mobile phase, stationary phase, method conditions, sample property, detection, and diluent/sample preparation procedures. The Ishikawa fishbone diagram demonstrates the principal risks and their associated sub-risks. All the probable main risks were thoroughly examined during the screening and optimization processes. A cause-and-effect "Ishikawa" fishbone diagram is shown in Supplementary Fig. 2.

### Screening of the critical method parameters (CMPs) using D-optimal split-plot design

Several factors may affect the elution and separation of peaks in the chromatographic technique. A constructive screening process is essential to eliminate the factors that have no or minimal impact. The screening of CMPs can be done using fractional factorial, full factorial, Plackett–Burman, Taguchi, and Split-Plot optimal designs. In the current study, we aimed to screen the six acidic buffers to choose the most suitable buffer as mobile phase-A; it is possible only with the D-optimal split-plot design.

The pKa of MPM (5.6 for the morpholino group and 8.5 for the phenolic group) is the origin of selecting the aqueous phase buffer. In the current study, we have chosen acidic buffer as mobile phase-A; those were 0.1% formic acid, 25 mM KH_2_PO_4_, 25 mM NaH_2_PO_4_, Water, 25 mM HCOONH_4_, and 25 mM CH_3_COONH_4_ by adjusting the pH to 3.2 with NaOH and OPA solutions as needed. A combination of organic solvent (Methanol or ACN) and water in the ratio of 70:30 (v/v) was chosen as mobile phase-B. Introduced water to the organic solvent to avoid the pumping issues associated with the pure organic solvent. A linear gradient program with a different initial composition of mobile phase-A: mobile phase-B such as 60:40 (v/v) and 80:20 (v/v); flow rates 0.3 and 0.5 mL min^−1^; and column oven temperatures 30 and 50 °C were chosen for the evaluation. Waters Acquity UPLC BEH C_18_ column was used as a stationary phase. A screening D-optimal split-plot design was created with 44 experimental runs. A mixture of the solution containing all the impurities and MPM was used to screen the CMPs.

The initial screening design experimental runs were unsuccessful in running because of the elevated column back pressure at higher flow rates. Methanol tends to generate higher back pressure as compared to other organic solvents. A new D-optimal split-plot design was created with 32 experiments by eliminating the methanol. The chromatographic runs were successful without having any column backpressure and pumping issues.

The number of peaks separated with a minimum resolution of 1.5 (Rs > 1.5) (R1), resolution between Imp-H and MPM (R2), resolution between MPM and Imp-D (R3), resolution between Imp-D and Imp-F (R4), and retention time of the late eluting peak in the chromatogram (R5) were the studied responses. The responses were carefully processed and fed into the statistical analysis tool. The 2FI model was selected for all responses and carefully analyzed. The screening D-optimal split-plot design data is shown in Supplementary Table 1.

**Figure 1:**
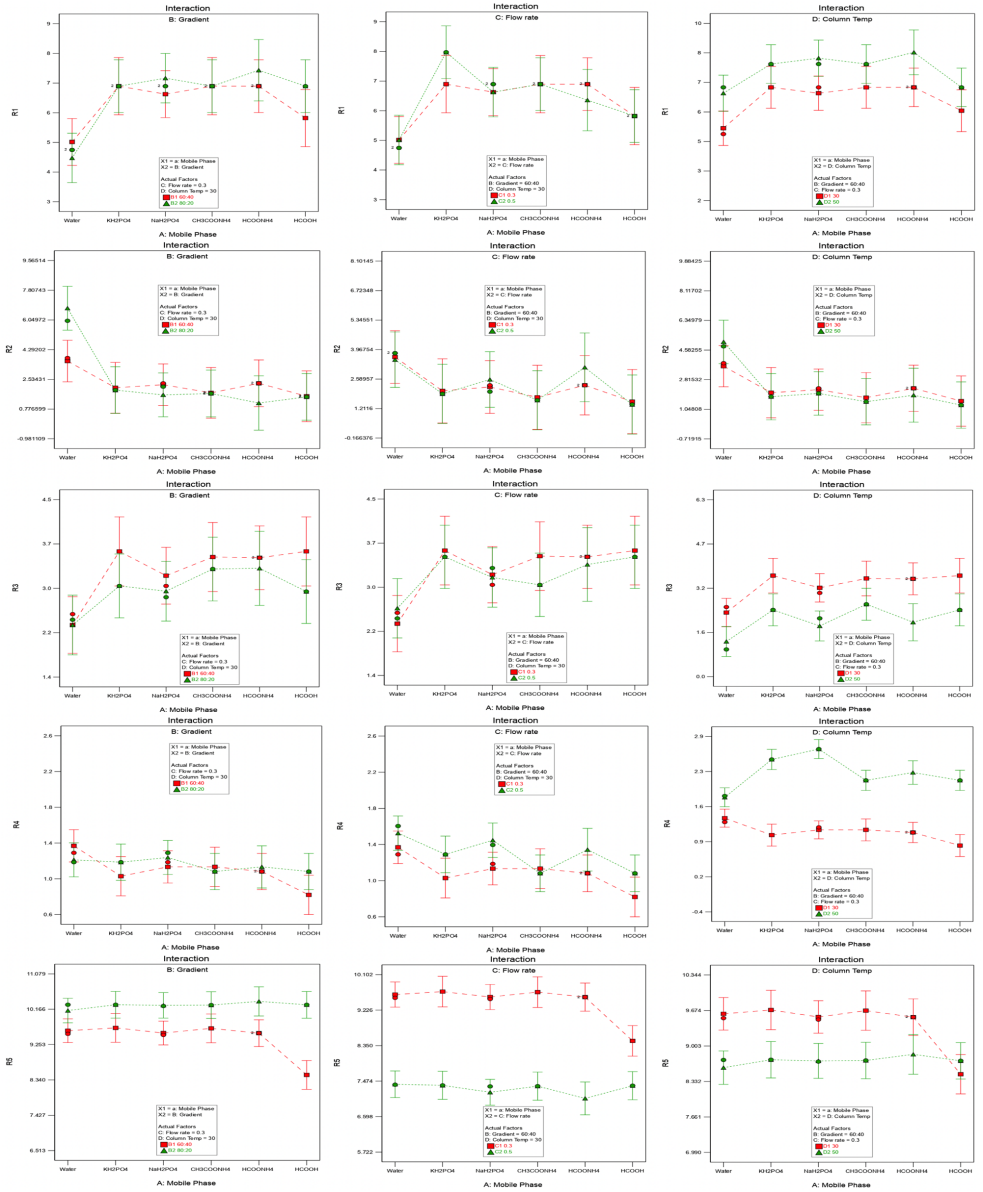
Interaction effect plots from the screening D-optimal split-plot design. R1: Number of impurity peaks separated with a minimum resolution of 1.5; R2: Rs between Imp-H and MPM; R3: Rs between MPM and Imp-D; R4: Rs between Imp-D and Imp-F; and R5: Rt of the late eluting peak.

More number of peaks (R1:8) were separated with a combination of KH_2_PO_4_ buffer and a higher flow rate (0.5 mL min^−1^); less number of peaks (R1:5) were separated with a combination of water and a higher percent of mobile phase-A (80%). The resolution between Imp-H and MPM (R2) was maximum (6.7) with a combination of water and a higher percent of mobile phase-A (80%); minimum (1.1) with a combination of HCOONH_4_ and a higher percent of mobile phase-A (80%). The resolution between MPM and Imp-D (R3) was maximum (3.6) with a combination of KH_2_PO_4_ buffer and a lower percent of mobile phase-A (60%); minimum (1.3) with a combination of water and higher column temperature (50 °C). The resolution between Imp-D and Imp-F (R4) was maximum (2.7) with a combination of NaH_2_PO_4_ buffer and higher column temperature (50 °C); minimum (0.8) with a combination of 0.1% HCOOH and lower column temperature (30 °C). The retention time of the late eluting peak (R5) was maximum (10.4) with a combination of HCOONH_4_ and a higher percentage of mobile phase-A (80%); minimum (7.0) with a combination of HCOONH_4_ and higher flow rate (0.5 mL min^−1^). The interaction effect plots from the screening D-optimal design are depicted in Fig. 1.

Numerical optimization was employed to identify the most influencing CMPs from the screening D-optimal design. All input CMPs such as mobile phase (A), initial gradient composition (B), flow rate (C), and column oven temperature (D) were selected as within the "Range." Response R1 goal was set as "maximize" with a lower value of 3 and a higher value of 8. The remaining responses were chosen as "None ." The output parameters from the D-optimal design were 25 mM KH_2_PO_4_ buffer (Factor-A), initial gradient composition of mobile phase-A (80%) (Factor-B), flow rate (0.5 mL min^−1^) (Factor-C), and column oven temperature (50 °C) (Factor-D). The global desirability was 1.000, and the number of peaks separated with a minimum resolution, i.e., Rs > 1.5 (R1 = 8), resolution between Imp-H and MPM (R2 = 1.4), resolution between MPM and Imp-D (R3 = 2.7), resolution between Imp-D and Imp-F (R4 = 2.4), and retention time of the late eluting peak in the chromatogram (R5 = 7.9) were the output responses. The numerical optimization plots from the D-optimal split plot design are shown in Supplementary Fig. 3.

### Selection of buffer concentration, pH, and the analytical column

It is necessary to evaluate the effect of concentration and pH of the attained buffer (KH_2_PO_4_) from the screening design. The studied buffer concentrations were 25, 50, and 100 mM while maintaining the pH of the buffer at 3.2. The remaining CMPs such as mobile phase-B (70% ACN), flow rate (0.5 mL min^−1^), and column oven temperature (50 °C) were kept constant throughout the study. A mixture of impurities and MPM solution was used for the evaluation. The concentration of the buffer did not show any influence on the elution and separation of the peaks. Therefore, a less concentration (25 mM) of buffer was chosen to minimize precipitation and column back pressure during the gradient analysis.

Evaluation of the buffer pH was conducted using 25 mM KH_2_PO_4_ with various pHs such as 4.0, 5.0, and 6.0. The pH of the buffer significantly affected the elution and separation of the peaks. There was a predominant change in the elution order of the impurities. Nevertheless, all the peaks were separated well with a better peak shape when using the pH 4.0 buffer as mobile phase-A. A solution of 25 mM KH_2_PO_4_ pH adjusted to 4.0 with OPA solution was finalized for further study.

Since the pH of the buffer significantly impacted the elution and separation of the peaks, different column chemistry stationary phases are likely to show similar results. Hence decided to examine various selectivity Acquity UPLC columns such as BEH C_8_, BEH Phenyl, HSS T_3_, Protein BEH C_4_ (100 × 2.1) mm, 1.7 µm, and BEH C_18_ columns. The separation of the peaks was found to be better in the case of BEH C_8_, HSS T_3_, and BEH C_18_ columns. However, symmetrical and sharp peaks for all impurities were observed using the Acquity UPLC BEH C_18_ (100 × 2.1) mm, 1.7 µm column.

The overall screening study experiments dictate that the finalized conditions were 25 mM KH_2_PO_4_ buffer pH adjusted to 4.0 with OPA as mobile phase-A, 70% ACN as mobile phase-B, 0.5 mL min^−1^ as flow rate, 50 °C as column oven temperature, and an Acquity UPLC BEH C_18_ analytical column as stationary phase.

### Optimization of the critical method parameters (CMPs) using central composite design (CCD): a response surface methodology (RSM)

Response surface Methodology (RSM) is one of the most effective statistical tools for optimization, determining the individual and combined effects of CMPs on CMAs. The CCD is an RSM-based design and helps identify the method operable design region (MODR) by employing numerical and graphical optimization. The current research's finalized conditions from the screening study experiments were comprehensively studied using a CCD. The obtained column oven temperature from the D-optimal screening design was 50 °C, which is fairly high; thus, deciding to study it as a maximum during the optimization. The CMPs for the CCD were pH of the buffer (Mobile phase-A) as factor-A, initial gradient composition (%Mobile phase-A) as factor-B, a flow rate of the mobile phase as factor-C, and column oven temperature as factor-D. The low, center, and high values were chosen as pH 3.7, 4.0, and 4.3 buffers for factor-A; 65, 80, and 95% for factor-B; 0.4, 0.5, and 0.6 mL min^−1^ for factor-C; 30, 40, and 50 °C for factor-D. A three-center points design with a face-centered (Alpha = 1) axial point was created for the current optimization study. A total of 27 experimental designs comprise 16 factorial, 8 axial, and 3 center point conditions. Acetonitrile and water in the ratio of 70:30 (v/v) were used as mobile phase-B. A spiked sample containing MPM and its impurities with an injection volume of 5 µL was utilized for the UPLC analysis.

The spiked sample solution contains 8 impurities and an active drug analyte (MPM), a total of 9 components, which produces 8 resolutions among all peaks. The resolution between the critical pair peaks is the CMAs for the current study. Out of eight resolutions, five resolutions are found to be critical those are Rs between Imp-E & A (R1), Imp-D & MPM (R2), MPM & Imp-F (R3), Imp-F & B (R4), and Imp-B & C (R5). The minimum resolution between other peak pairs, such as Imp-A & H, Imp-H & D, and Imp-C & G were 4.3, 3.1, and 23.0, respectively. The zero (0) value indicated either coelution of the critical peak pairs or a change in the order of elution of the peaks. The obtained CCD data with input variables and output responses are tabulated in Table 2.

**Table 2:** The CCD data with input variables and output responses^a,b^.

Run	Factor 1	Factor 2	Factor 3	Factor 4	Response 1	Response 2	Response 3	Response 4	Response 5
	A: pH of the Buffer	B: Initial Gradient (% MP-A)	C: Flow Rate (mL/min)	D: Column Temperature (°C)	R1	R2	R3	R4	R5
1	4.3	65	0.6	30	5.8	5.1	2.2	1.2	3.9
2	4.0	80	0.4	40	8.1	4.0	4.2	1.3	7.1
3	4.0	65	0.5	40	7.5	3.6	3.4	0.0	5.8
4	4.3	80	0.5	40	1.6	7.0	0.0	2.6	0.0
5	4.0	80	0.5	40	6.5	3.4	0.0	4.1	6.7
6	3.7	65	0.4	30	13.8	0.0	3.6	6.5	6.6
7	4.0	95	0.5	40	6.9	3.7	0.0	3.6	6.1
8	4.0	80	0.5	30	9.2	1.4	3.8	2.6	6.1
9	3.7	65	0.4	50	10.5	1.7	4.4	3.0	7.7
10	4.0	80	0.5	50	2.8	6.0	0.0	4.2	3.6
11	3.7	95	0.4	30	13.5	0.0	3.7	7.7	6.7
12	4.3	65	0.4	30	7.3	5.4	0.0	2.7	4.7
13	4.3	95	0.6	30	4.9	5.0	1.7	1.4	3.8
14	3.7	95	0.6	30	10.8	0.0	3.5	4.2	6.5
15	4.3	95	0.4	50	0.0	10.1	0.0	0.0	2.3
16	4.3	95	0.6	50	0.0	5.9	0.0	0.0	2.8
17	4.3	95	0.4	30	8.2	5.6	2.3	0.0	4.9
18	4.3	65	0.4	50	1.5	9.7	0.0	0.0	0.0
19	3.7	95	0.4	50	10.6	1.9	4.5	2.9	7.8
20	3.7	95	0.6	50	7.1	1.2	0.0	3.9	7.3
21	4.0	80	0.6	40	5.8	3.0	3.2	1.1	6.2
22	4.3	65	0.6	50	0.0	0.0	0.0	0.0	3.0
23	3.7	65	0.6	50	8.6	1.4	0.0	0.0	7.5
24	4.0	80	0.5	40	10.1	3.3	0.0	4.1	6.8
25	3.7	65	0.6	30	14.1	0.0	3.4	4.2	6.5
26	3.7	80	0.5	40	15.3	0.0	4.0	3.2	7.3
27	4.0	80	0.5	40	9.9	3.3	0.0	4.1	6.7

^a^R1: Rs between Imp-E & A; R2: Rs between Imp-D & MPM; R3: Rs between MPM & Imp-F; R4: Rs between Imp-F & B; R5: Rs between Imp-B & C.

^b^Response value 0.0 indicates either coelution of the peaks or change in order of elution of the peaks.

The suggested best-fitting two-factor interaction (2FI) model was selected and analyzed for each response. The model is statistically significant for all responses (R1–R5), which refers to its suitability for the current study. The factors A, C, and D are the essential model terms for R1. Similarly, A, C, D, AC, and CD for R2; A and D for R3 and R4; and A and AD for response R5. The significance of model terms is shown in Supplementary Table 2. The 2FI equation was generated for each response and tabulated in Table 3. The equation can be adjusted by changing the CMP values to achieve the desired response.

**Table 3:** Two-factor interaction equation for CMAs^a^.

Coefficient code	Polynomial coefficients for CMA				
	R1	R2	R3	R4	R5
*Y*_*0*_	+ 63.91759	-68.16667	+ 52.54907	+ 72.09352	+ 14.84815
*Y*_*A*_	− 11.59028	+ 17.41204	− 16.14120	− 11.06713	− 1.28241
*Y*_*B*_	− 0.130324	− 0.534815	+ 0.003935	+ 0.235509	− 0.076296
*Y*_*C*_	− 6.02778	+ 124.66667	− 60.75000	− 97.41667	− 23.66667
*Y*_*D*_	+ 0.483958	+ 0.449722	+ 0.368958	− 1.00854	+ 0.840972
*Y*_*AB*_	+ 0.048611	+ 0.088889	+ 0.020833	− 0.104167	+ 0.030556
*Y*_*AC*_	+ 3.12500	− 28.75000	+ 22.70833	+ 16.04167	+ 5.41667
*Y*_*AD*_	− 0.193750	− 0.033333	− 0.018750	+ 0.156250	− 0.275000
*Y*_*BC*_	− 0.204167	+ 0.200000	− 0.120833	+ 0.237500	− 0.133333
*Y*_*BD*_	+ 0.000292	+ 0.002583	− 0.000792	+ 0.002125	+ 0.000750
*Y*_*CD*_	+ 0.018750	− 0.875000	− 0.631250	+ 0.243750	+ 0.312500

^a^R1: Rs between Imp-E & A; R2: Rs between Imp-D & MPM; R3: Rs between MPM & Imp-F; R4: Rs between Imp-F & B; R5: Rs between Imp-B & C.

**Figure 2:**
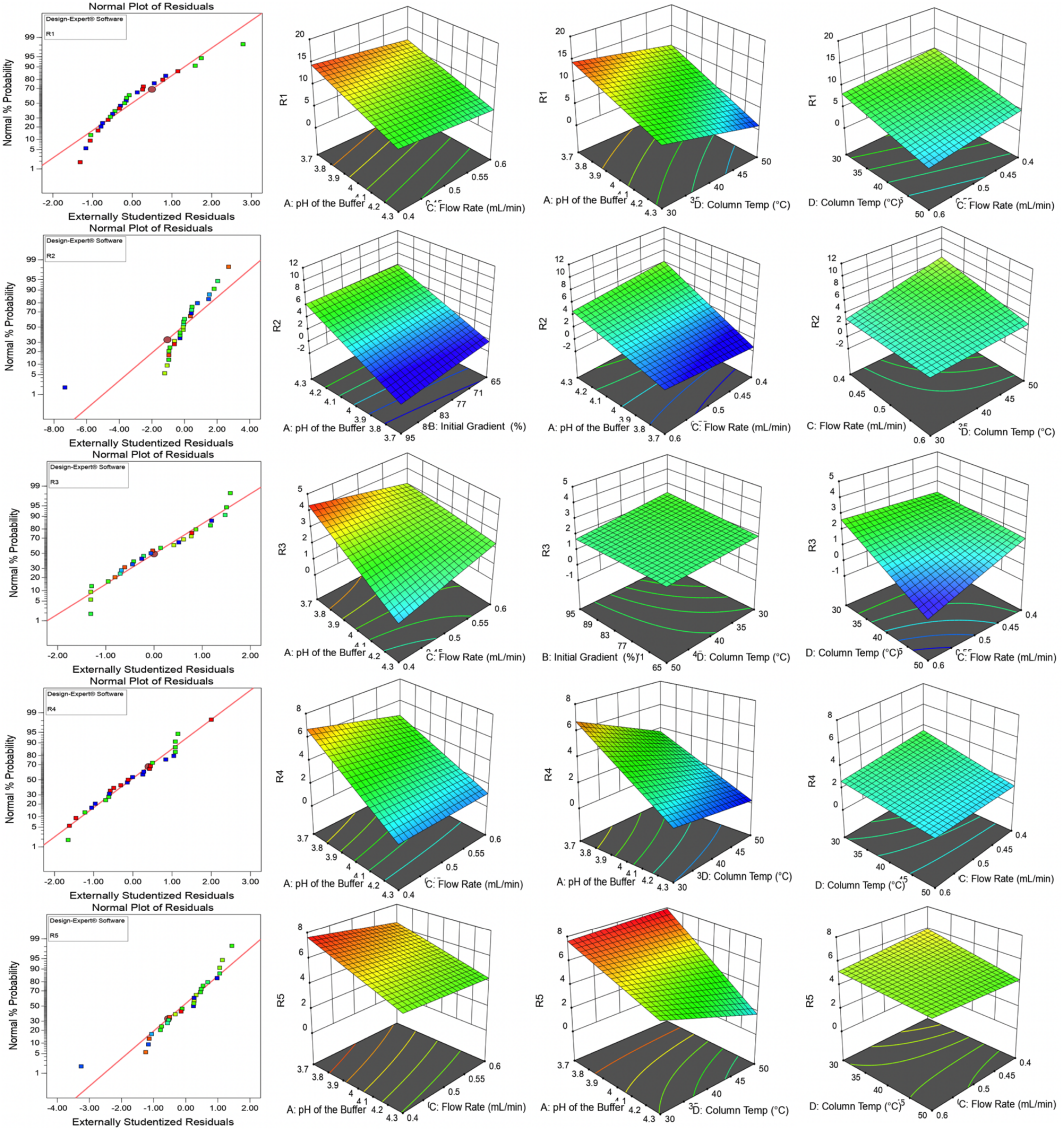
2D-Normal plots and 3D-Response surface plots for the responses (R1–R5). R1: Rs between Imp-E & A; R2: Rs between Imp-D & MPM; R3: Rs between MPM & Imp-F; R4: Rs between Imp-F & B; R5: Rs between Imp-B & C.

The resolution between Imp-E & A (R1) was maximum (14.9653) at lower pH (3.7), lower initial gradient composition (65%), lower flow rate (0.4 mL min^−1^), and minimum (5.05833) at all high conditions. The resolution between Imp-D & MPM (R2) was maximum (6.27963) at higher pH (4.3), higher initial gradient composition (95%), and lower flow rate (0.4 mL min^−1^); minimum (− 1.73426) at lower pH (3.7), higher initial gradient composition (95%), and lower flow rate (0.4 mL min^−1^). The resolution between MPM & Imp-F (R3) was maximum (4.3963) at lower pH (3.7), higher initial gradient composition (95%), and lower flow rate (0.4 mL min^−1^); minimum (0.368519) at higher pH (4.3), softer initial gradient composition (65%), and lower flow rate (0.4 mL min^−1^). The resolution between Imp-F & B (R4) was maximum (6.77269) at lower pH (3.7), higher initial gradient composition (95%), and lower flow rate (0.4 mL min^−1^); minimum (0.857407) at higher pH (4.3), higher initial gradient composition (95%), and lower flow rate (0.4 mL min^−1^). The resolution between Imp-B & C (R5) was maximum (7.67037) at lower pH (3.7), higher initial gradient composition (95%), and lower flow rate (0.4 mL min^−1^); minimum (4.25926) at higher pH (4.3), higher initial gradient composition (95%), and higher flow rate (0.6 mL min^−1^). The 2D-normal plots and 3D-response surface plots for the responses (R1–R5) are shown in Fig. 2.

**Figure 3:**
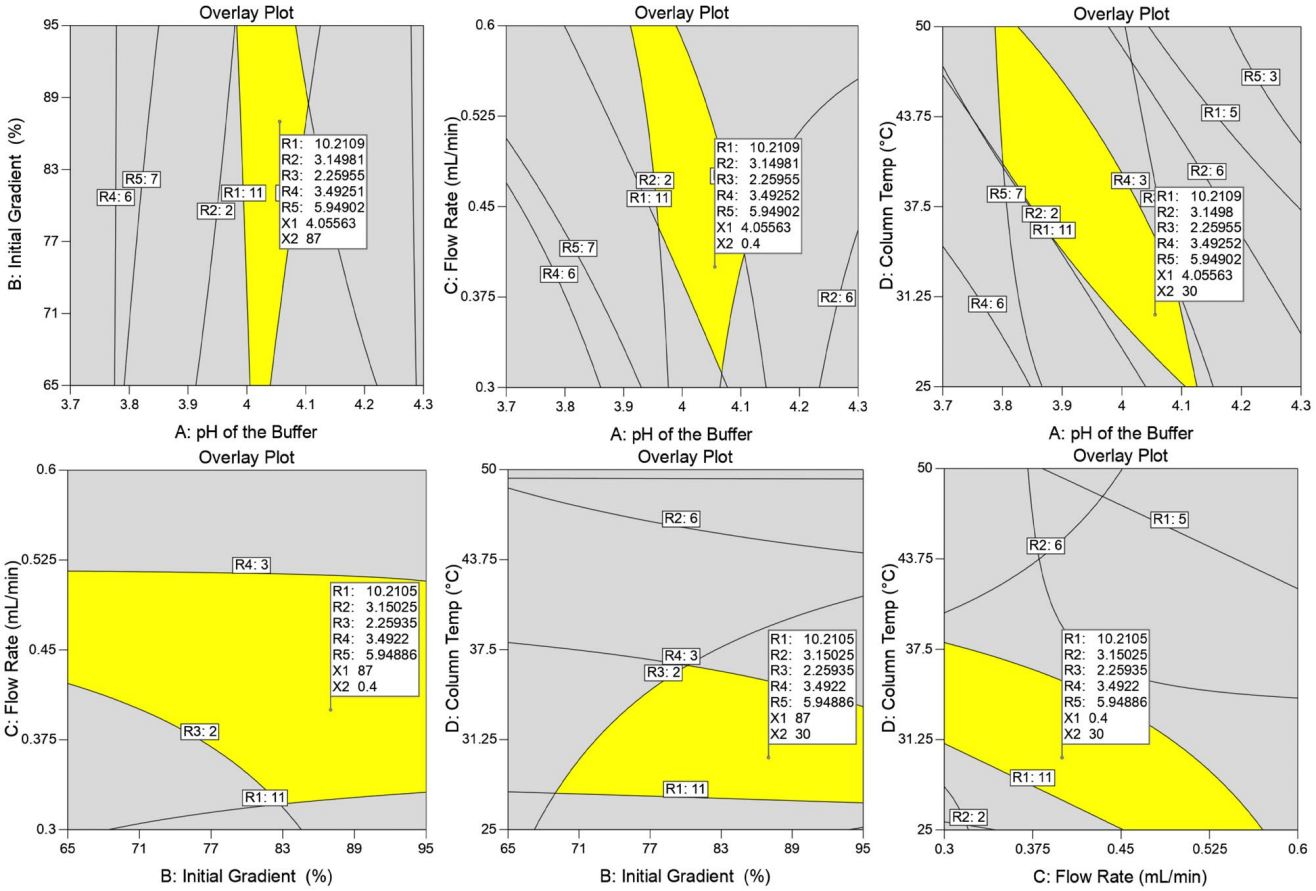
The overlay plots from the graphical optimization.

Numerical optimization is a process of identifying the most opportune CMPs for the robust analytical method. The input parameter such as pH of the buffer (CMP-A) was set as in "Range," initial gradient composition (CMP-B) was set as "Equal to" with a value of 87, flow rate (CMP-C), and column oven temperatures (CMP-D) was set as "Minimize" with the importance of high (+ +  +  + +). In the present study, organic solvent (ACN) is a hazardous chemical. CMP-B and CMP-C were set following the green chemistry principle of minimizing dangerous chemicals. The set goal for the responses (R1–R5) was maximized because the resolution between the critical peaks is coveted to be high. The outcome of the CCD was pH of the buffer (CMP-A) was 4.05563, the initial gradient composition (CMP-B) was 87%, the flow rate of the mobile phase (CMP-C) was 0.4 mL min^−1^, and the column oven temperature was 30 °C. The output CMAs such as R1 = 10.2109, R2 = 3.14981, R3 = 2.25955, R4 = 3.49251, and R5 = 5.94902. The predicted output resolutions with the proposed CMPs were substantially higher than 2.0, indicating an excellent separation between the critical peak pairs. A confirmatory analysis was conducted by employing the recommended input variables, and the obtained output CMAs matched the predicted responses. The solution from the numerical optimization from the CCD is shown in Supplementary Fig. 4. The design exhibited superior desirability of 0.968, indicating that the method is applicable globally at about 96.8%. The contour plots of the desirability and the predicted responses are shown in Supplementary Fig. 5. The global desirability plots from the numerical optimization are shown in Supplementary Fig. 6. A graphical optimization was conducted to identify the method operable design space region and mark the responses. The overlay plots from the graphical optimization are shown in Fig. 3.

### Identification of method operable design space region (MODR)

Identifying an operable design space region is essential when developing QbD-based methods. MODR helps to identify the analytical method's control strategy and robust method conditions. The current process MODR was considered from the numerical optimization, graphical optimization, and overlay plots. The developed method MODR for CMP-A is pH 3.9–4.1, CMP-B is 70–95%, CMP-C is 0.4–0.45 mL min^−1^, and CMP-D is 30–37 °C.

### Optimization of diluent and sample preparation

Selection of the most suitable diluent and establishing a sample preparation procedure are vital while achieving sample recovery. All desired analytes should be extracted completely into the diluent, and they must be stable. Diluent selection depends on the solubility of the analytes and the inactive ingredients of the formulated drug product. The current studied formulation product is a powder for oral suspension, which means the ingredients present in the powder and suspension formulation after reconstitution with water. The active drug is insoluble in water; hence, introducing an organic solvent is essential to dissolve them.

Initially, 100% organic solvents such as acetonitrile and methanol were used to extract the analytes from the sample matrix. Equivalent to 1.0 mL of suspension sample was weighed into a 100 mL volumetric flask, dissolved and diluted to volume with an organic solvent, and mixed well. The suspension was stuck at the bottom and neither dissolved nor dispersed. The incomplete extraction of the analyte caused less recovery of the impurities. Hence, water and ACN 50:50 (v/v) was used as an extraction medium and diluent; the sample's complete dispersion with most of the ingredients is solubilized. The resultant chromatogram showed an increase in the response of the active drug and the impurities. The recoveries of the contaminants were found to be > 95%. However, the peak shape of the impurities was slightly distorted due to a higher percentage of organic solvent. Further, the sample solutions were prepared using water and ACN in the ratio of 80:20 (v/v) for extraction and dilution. The resultant chromatogram showed an excellent and symmetrical peak shape with a recovery of > 95%.

The stability of the sample solution in various diluents was assessed to identify the suitable diluent. The water: ACN (80:20) (v/v), 0.1% formic acid in water: ACN (80:20) (v/v), and mixture of KH_2_PO_4_ buffer (pH 3.0 or 4.0 or 5.0) and ACN (80:20) (v/v) were used to prepare the sample solution. The prepared solutions were injected at various time intervals, such as initial, 24 h, and 48 h, and calculated the %difference of the impurities from the initial. The MPA (Imp-C) was increased significantly in water (> 200%), pH 4.0 buffer (> 30%), and pH 5.0 buffer (> 50%) samples over 48 h. At the same time, 0.1% formic acid and pH 3.0 buffer solution samples were not shown such variation (< 6%). These variations were not observed in the case of any other impurities. It indicates that the MPA is formed at higher pH (Basic) conditions; hence we have chosen 0.1% formic acid in water and ACN in the ratio of 80:20 (v/v) as an extraction medium and diluent for the current study. The final sample solution concentration is 100 µg mL^−1^ of MPM was achieved by taking 1 mL of suspension into 100 mL VF, extracting, and diluting to volume with diluent, further diluting 5.0 mL to 100 mL with diluent. The appropriate response of the analyte and the impurities were observed with an injection volume of 5.0 µL.

### Method validation

The obtained method conditions such as eluents, analytical column, gradient program, initial gradient composition, flow rate, column temperature, diluent, and sample preparation procedure were considered from optimization experiments (as mentioned in “Materials and methods” section) to validate the test method.

### System suitability

The analytical method's suitability was demonstrated by injecting the standard solution six times. The tailing factor, theoretical plates, and %CV were determined. In the entire validation study, the leading tailing factor, minimum theoretical plates, and maximum %CV were 1.2, 91,119, and 3.3, respectively. The data implies that the system is suitable for the current study.

### LOD and LOQ

The analytical method sensitivity was established by determining the LOD and LOQ of analyte peaks. LOD and LOQ values were computed using the signal-to-noise ratio of the 10% linearity solution, which contains all impurities and MPM. The signal-to-noise ratios for the LOD and the LOQ concentrations were set to 3:1 and 10:1, respectively. The obtained LOD and LOQ concentrations are presented in Table 4.

**Table 4:** Method validation data.

Test	Details	Acceptance Criteria	Results								
			Imp-E	Imp-A	Imp-H	Imp-D	MPM	Imp-F	Imp-B	Imp-C	Imp-G
Specificity	Retention time *n* = 1, each impurity at 1.0 µg mL^−1^	Each peak should elute at a different retention time	4.953	5.418	6.137	6.509	6.871	7.146	7.289	7.658	9.820
			There are no peak interferes with placebo peaks. All the individual impurity peaks eluted at different retention times								
Linearity	Range (µg mL^−1^)	Range (µg mL^−1^)	0.0371–0.7545	0.0321–0.7321	0.0233–0.7508	0.0467–0.7509	0.0333–0.7497	0.0293–0.7678	0.0363–0.7584	0.0347–4.9634	0.0436–0.7636
	Minimum 6 concentration levels in the range LOQ–150% of specification	Correlation coefficient should be > 0.999	0.999	0.999	0.999	0.999	0.999	0.999	0.999	0.999	0.999
	Slope		44,357.242	60,717.374	138,890.406	66,685.223	71,979.649	86,647.962	90,671.303	101,629.307	83,134.619
	Intercept		1759.467	58.533	− 237.267	− 501.467	1041.667	− 82.800	− 121.667	− 770.800	− 475.467
	STEYX SD		520.919	121.679	264.279	515.648	71.593	229.146	390.461	719.195	283.291
Limit of detection	0.01 µg mL^−1^ (lowest detectable concentration)	S/N ratio > 3	0.011	0.010	0.007	0.014	0.010	0.009	0.011	0.010	0.013
Limit of quantitation	0.03 µg mL^−1^ (lowest quantitatable concentration)	S/N ratio > 10	0.037	0.032	0.023	0.046	0.033	0.029	0.035	0.034	0.043
Accuracy	*n* = 9 (3 determinations each at LOQ, 50%, 100% and 150% specification level	Recovery at each level should be 90–110%	99.8 ± 1.1 100.2 ± 3.0 98.0 ± 2.1 99.7 ± 3.0	100.6 ± 2.6 101.0 ± 3.2 98.2 ± 0.8 102.4 ± 2.1	102.1 ± 2.9 100.5 ± 3.8 99.7 ± 2.0 100.7 ± 2.4	98.5 ± 2.5 98.2 ± 3.1 98.0 ± 2.2 98.1 ± 1.2	102.7 ± 2.0 100.1 ± 2.9 99.9 ± 0.5 100.5 ± 3.3	96.2 ± 1.9 96.7 ± 1.0 99.3 ± 0.5 98.2 ± 1.9	99.8 ± 2.3 100.3 ± 2.9 98.3 ± 2.3 101.5 ± 2.6	98.9 ± 3.8 100.6 ± 2.1 97.8 ± 1.2 100.0 ± 2.9	98.4 ± 1.3 101.4 ± 1.7 101.3 ± 2.5 99.5 ± 1.7
Precision (Analyst 1)	*n* = 6 (6 determinations each at 1.0 µg mL^−1^)	RSD should be < 5%	1.6%	2.5%	2.8%	2.8%	2.2%	1.6%	3.0%	1.5%	2.1%
Intermediate precision (Analyst 2)	*n* = 6 (6 determinations at µg mL^−1^)	RSD should be < 5%	4.5%	0.8%	1.6%	2.1%	3.3%	1.9%	1.2%	1.8%	2.2%

### Linearity

The impact of concentration was demonstrated by preparing solutions of the impurities and MPM (LOQ to 150% of the specification level) and analyzing. By plotting the concentration versus the peak area data generated a linear regression equation for each analyte. All analytes showed an excellent linear response from LOQ to 150% of specification, and the correlation coefficients were > 0.999. The relative response factors (RRF) were determined from the slope of the impurity and MPM. The results from the linearity experiment are presented in Table 4.

### Specificity

**Figure 4:**
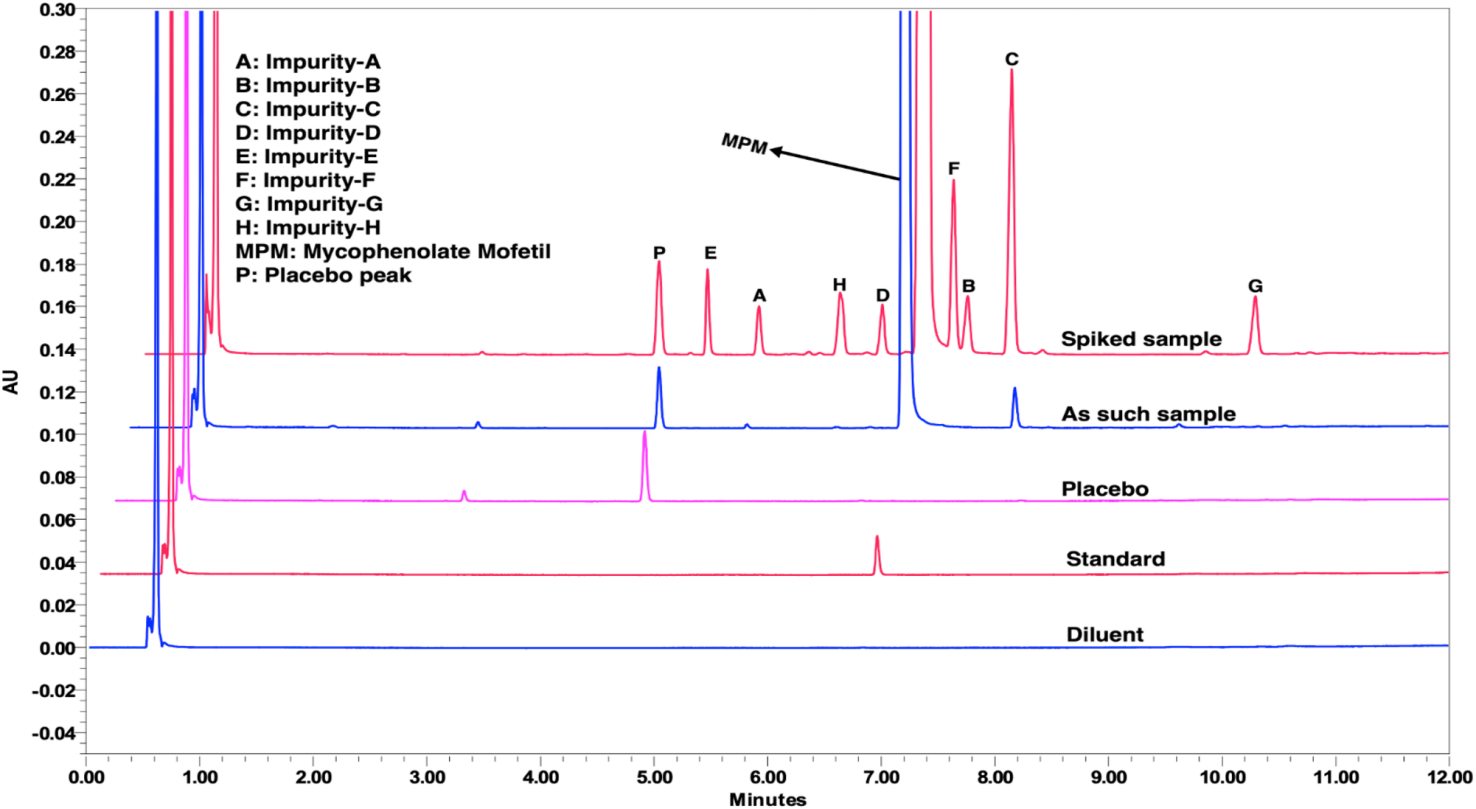
Interference study chromatograms from the final method. Chromatographic conditions: MP-A: pH 4.05 KH_2_PO_4_ (25 mM); MP-B: Water: ACN = 30:70 (v/v); Initial gradient composition MP-A:MP-B (87:13); Column: Acquity UPLC BEH C18 (100 mm × 2.1 mm), 1.7 µm; Flow rate: 0.4 mL min^−1^; Column temperature: 30 °C; Injection volume: 5 µL; and Wavelength detection: 215 nm.

The analytical method specificity was demonstrated by injecting a diluent, placebo, standard, as such sample, a spiked sample with impurities and individual impurities into the UPLC system. The final chromatograms showed that the placebo peaks did not interfere with the impurities and MPM. The peak purity of all components was passed without any flag. The interference study chromatograms are displayed in Fig. 4, and the results are tabulated in Table 4.

**Figure 5:**
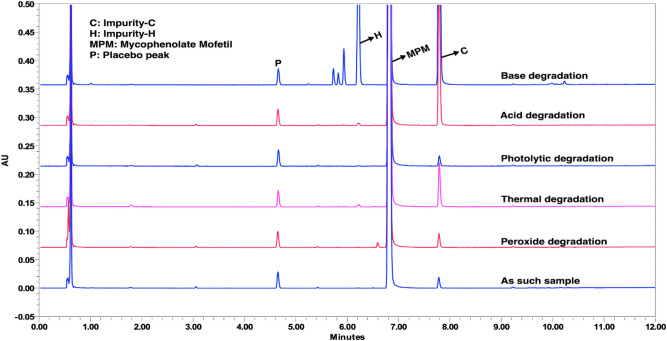
An overlaid chromatogram from the stress study experiments. Chromatographic conditions: MP-A: pH 4.05 KH_2_PO_4_ (25 mM); MP-B: Water: ACN = 30:70 (v/v); Initial gradient composition MP-A:MP-B (87:13); Column: Acquity UPLC BEH C18 (100 mm × 2.1 mm), 1.7 µm; Flow rate: 0.4 mL min^−1^; Column temperature: 30 °C; Injection volume: 5 µL; and Wavelength detection: 215 nm.

The sample was subjected to various stress conditions to demonstrate the stability-indicating ability of the analytical method. The exposed state for acid degradation was 1.0 M HCl/1 mL/24 h at RT, base degradation was 1.0 M NaOH/1 mL/24 h at RT, oxidative degradation was 6% H_2_O_2_/1 mL/24 h at RT, and thermal degradation was 70 °C for 24 h. The photolytic degradation conditions were followed as per ICH Q1B recommendations. The major degradant formed in acid degradation was impurity-C. In base degradation, two known impurities (impurity-C and H) and three unknown impurities were formed. In oxidative degradation, impurity-C and D were formed. In thermal degradation, only impurity-C was observed. No degradation was observed in the case of photolytic degradation. Few unknown impurities at RRT 0.84, 0.85, and 0.87 were observed from the base degradation study and eluted between impurity-A and H without interference. The overlaid chromatograms of stress studies are shown in Fig. 5.

The overall degradation study indicated that the MPM is sensitive to acidic and basic conditions and forms Imp-C as a major degradant. The peak purity results showed that the MPM peak is homogenous. The outcome of the degradation study has proved that the analytical method is stability-indicating. The % degradation and peak purity results are presented in Supplementary Table 3.

### Identification of unknown impurities formed in base degradation

Base degradation sample analysis was carried out using the LCMS method described in the “Materials and methods” section. The analysis type was selected as accurate mass screening on MS^E^ data to identify MS and MSMS peaks. The elution order of the peaks in the chromatogram was similar to the original UPLC method. Following analysis of the individual solution chromatograms, Imp-H (sorbitol ester of MPA), MPM, and MPA were eluted at retention times of 8.56, 9.60, and 10.76 min, respectively. The acquired total ion mass chromatograms for both positive and negative modes are presented in Supplementary Fig. 7(a, b). The LCMS data was processed using scientific libraries from NIST and chem spider.

**Figure 6:**
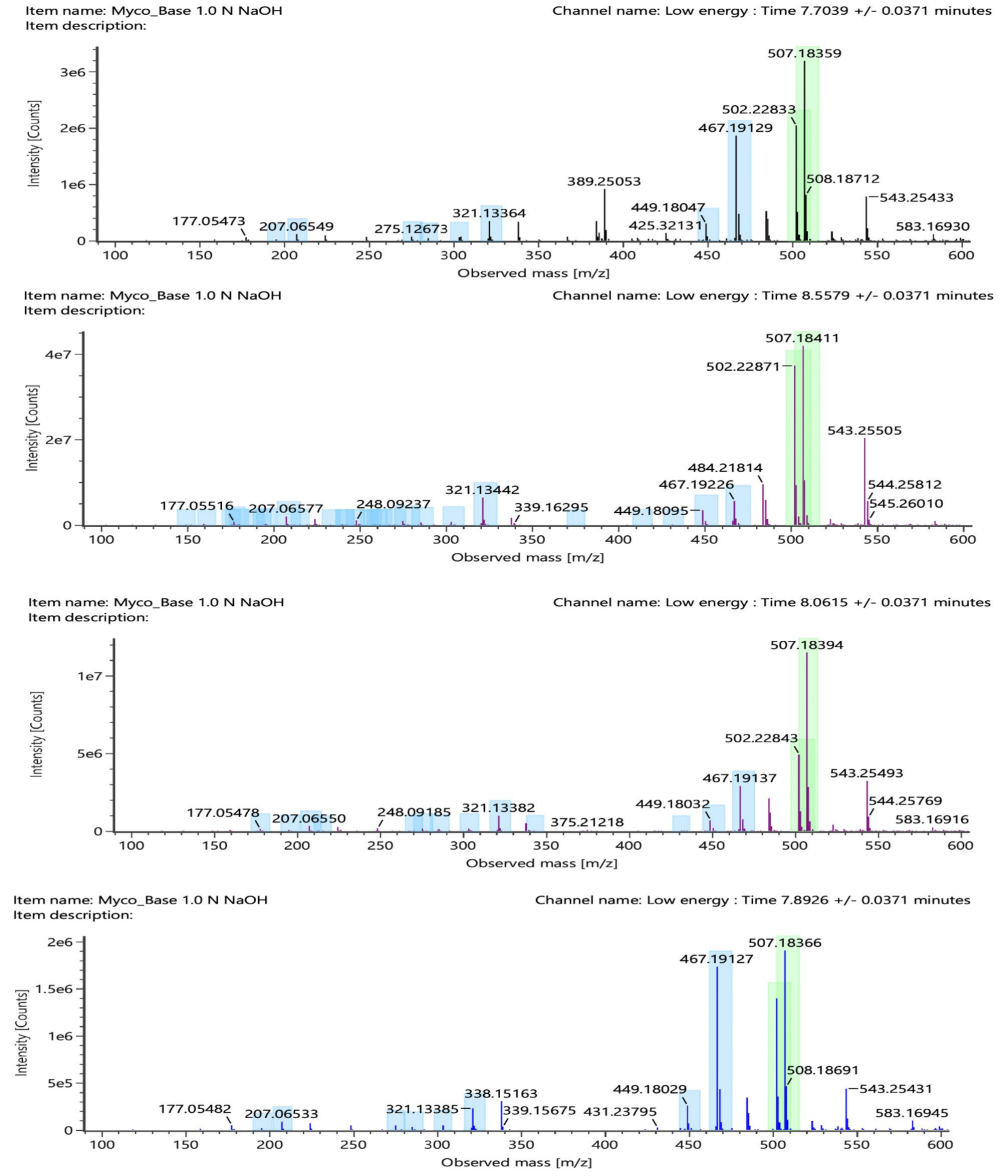
Positive mode low energy mass spectrums (**a**) Unknown at RRT 0.84; (**b**) Unknown at RRT 0.85; (**c**) Unknown at RRT 0.87; (**d**) Mycophenolic acid sorbitol ester.

Upon carefully reviewing the MS and MS^E^ data, we identified the molecular and fragment ion peaks. Molecular ion peaks in the positive mode analysis for MPM and MPA were 434.2 [M + H]^+^, 456.2 [M + Na]^+^; and 321.1 [M + H]^+^, 338.2 [M + NH_4_]^+^, 343.1 [M + Na]^+^, respectively. The molecular ion peaks in positive mode for Imp-H and the unknown impurities at Rt 7.70, 7.89, and 8.05 exhibited the same m/z values as 502.2 [M + NH_4_]^+^ and 507.2 [M + Na]^+^. Similarly, molecular ion peaks in the negative mode analysis for MPM and MPA were 432.2 [M − H]^–^ and 319.1 [M − H]^–^, respectively. The molecular ion peaks in the negative mode for Imp-H and the unknown impurities at Rt 7.70, 7.89, and 8.05 exhibited the same m/z value 483.2 [M − H]^–^. The mass spectrums are shown in Fig. 6 and Supplementary Fig. 88–11. The observed molecular and major fragment ion peaks in positive and negative mode analysis are tabulated in Table 5.

**Table 5:** Molecular and fragment ions peaks in positive and negative ionization mode.

S. no.	Name of the peak	Positive mode (m/z values)		Negative mode (m/z values)	
		Molecular ion peaks (Low collision energy)	Fragment ion peaks (High collision energy)	Molecular ion peaks (Low collision energy)	Fragment ion peaks (High collision energy)
1	RT 7.70	507.18359 [M + Na]^+^, 502.22833 [M + NH_4_]^+^	467.19241, 449.18087, 343.11522, 321.13318, 303.12380, 285.11280, 275.12820, 229.04785, 207.06568, 195.06490	483.18785 [M − H]^–^	341.10144, 319.11937, 301.10860, 287.09036, 275.12925, 269.08292, 207.06604, 191.03611
2	RT 7.89	507.18366 [M + Na]^+^, 502.22819 [M + NH_4_]^+^	467.19241, 449.18199, 343.11497, 321.13428, 303.12389, 285.11257, 275.12835, 229.04739, 207.06574, 195.06533	483.18781 [M − H]^–^	341.10124, 319.11929, 301.10784, 287.09376, 275.12922, 269.08347, 207.06505, 191.03552
3	RRT 8.05	507.18394 [M + Na]^+^, 502.22843 [M + NH_4_]^+^	467.19221, 449.18159, 343.11562, 321.13409, 303.12392, 285.11281, 275.12857, 229.04697, 207.06586, 195.06513	483.18779 [M − H]^–^	341.10100, 319.11952, 301.10903, 287.09215, 275.12946, 269.08354, 207.06445, 191.03569
4	Imp-H (Mycophenolic acid sorbitol ester)	507.18411 [M + Na]^+^, 502.22871 [M + NH_4_]^+^	467.19312, 449.18223, 343.11616, 321.13446, 303.12423, 285.11304, 275.12892, 229.04739, 207.06596, 195.06549	483.18737 [M − H]^–^	341.10092, 319.11932, 301.10788, 287.09276, 275.12914, 269.08243, 207.06515, 191.03538
5	Imp-C (MPA)	343.11636 [M + Na]^+^, 338.16098 [M + NH_4_]^+^, 321.13451 [M + H]^+^	303.12495, 275.12954, 229.04750, 207.06646, 195.06633, 159.04514	319.11855 [M − H]^–^	287.09244, 275.12891, 269.08172, 244.06643, 207.06590, 191.03523, 179.03514
6	Active drug (MPM)	456.20026 [M + Na]^+^, 434.21818 [M + H]^+^	347.15017, 303.12417, 285.11384, 207.06655, 195.06633, 159.04457, 114.08877	432.20233 [M − H]^–^	400.17589, 371.13593, 301.10867, 269.08167, 245.08152, 217.04763, 191.03482

The positive and negative MS and MS^E^ spectrums showed similar molecular and fragment ion peaks for the unknowns at Rt 7.70, 7.89, and 8.05, as well as imp-H. It indicates that the unknown peaks at Rt 7.70, 7.89, 8.05, and Imp-H are similar in structure. Furthermore, the UV spectral data of unknown degradation impurities and impurity-H are the same, confirming that the unknown degradation impurities are structural isomers of impurity-H. The positive and negative modes at low and high collision energies data demonstrate that the peaks at Rt 9.64 and 10.75 are the MPM and its active metabolite MPA, respectively.

**Figure 7:**
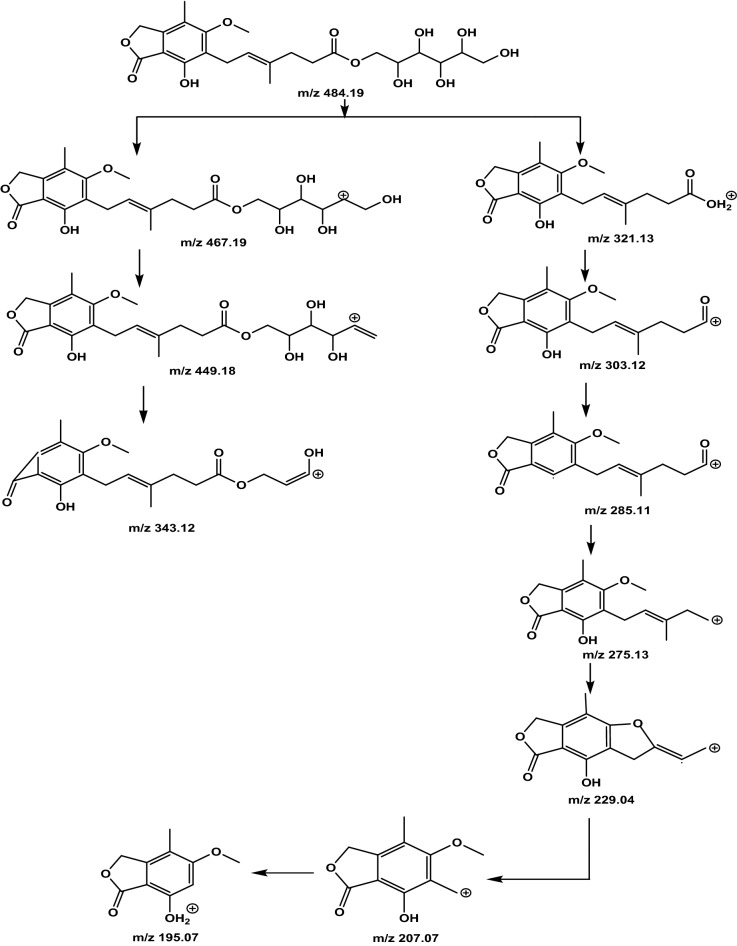
Fragmentation pattern of sorbitol ester of mycophenolic acid and its isomers.

The MPA has two isomeric forms: E and Z. Similarly, sorbitol has isomeric forms, D and L. A combination of two isomeric forms of MPA and sorbitol produces 4 isomeric ester impurities; while other forms are predominant, Z-MPA and L-sorbitol are present in trace amounts. The corresponding esters are also formed in less quantity. According to our understanding, the peaks at Rt 7.70 and 7.89 are a combination of Z-MPA and D & L-Sorbitol esters. The peak at Rt 8.05 is an ester of E-MPA and L-Sorbitol. The known impurity-H is formed from E-MPA and D-Sorbitol. The observed impurity levels were 0.6, 0.4, 1.5, and 4.0% and eluted at 7.70, 7.89, 8.05, and 8.55 min, respectively. The fragmentation pattern of sorbitol ester of mycophenolic acid and its isomers is shown in Fig. 7.

### Precision

Six spiked samples were prepared at the specification level and analyzed by two analysts on different days to demonstrate the method's precision. Calculated the % RSD for both the analysts and found they ranged from 1.5 to 3.0 and 0.8 to 4.5 from analysts 1 and 2, respectively. The results dictate that the method is precise for determining the impurities in MPM and its oral suspension formulation. The precision results are presented in Table 4.

### Accuracy

The method's accuracy was executed on a control sample by spiking the impurities at LOQ to 150% of the specification level. Samples were prepared in triplicate for each concentration. Calculated the % recovery for each group; the results ranged from 94.7 to 104.8%. A summary of the recovery results is provided in Table 4.

### Robustness

The robust method conditions for the developed method were considered from the MODR of CCD. The developed method is robust within the MODR. The robust sample preparation conditions were executed by varying the sonication time ± 5 min from the actual method condition (15 min). The variations in method conditions did not influence the results. The developed method is proven to be robust.

### Solution stability

The standard and sample solutions stability was evaluated at room and refrigerator temperatures. The standard and sample solutions are stable up to 48 h at refrigerator storage conditions and unstable at room temperature. Hence sample solutions shall be stored under refrigerator conditions.

### Method greenness assessment

The current study used GAPI, AGREE, and analytical eco-scale tools to assess the method's greenness. The total run time is 15 min with a flow rate of 0.4 mL min^−1^, and it consumes less than 5.0 mL of organic solvent (ACN). 40.0 mL of ACN is required for each sample preparation, and a sum of < 50 mL ACN is consumed for each sample analysis. The total penalty points for the method are 15. The eco-scale score for this analytical method is 85, indicating the current process's excellent greenness. GAPI pictograms represent all aspects of the method in each stage, and they are filled with green, yellow, and red colors. It incorporates all the stages of the analysis into a single pictogram with colors, which is a new advanced tool for evaluating greenness from all angles. The 15 small pictograms show the method's greenness; out of 15, only one pictogram, which is solvents/reagents used for analysis in the red region, remained pictograms indicating (yellow to green) the method's greenness. The Gdask University of Technology, Poland, developed analytical greenness (AGREE), another tool based on the principles of green analytical chemistry.

**Figure 8:**
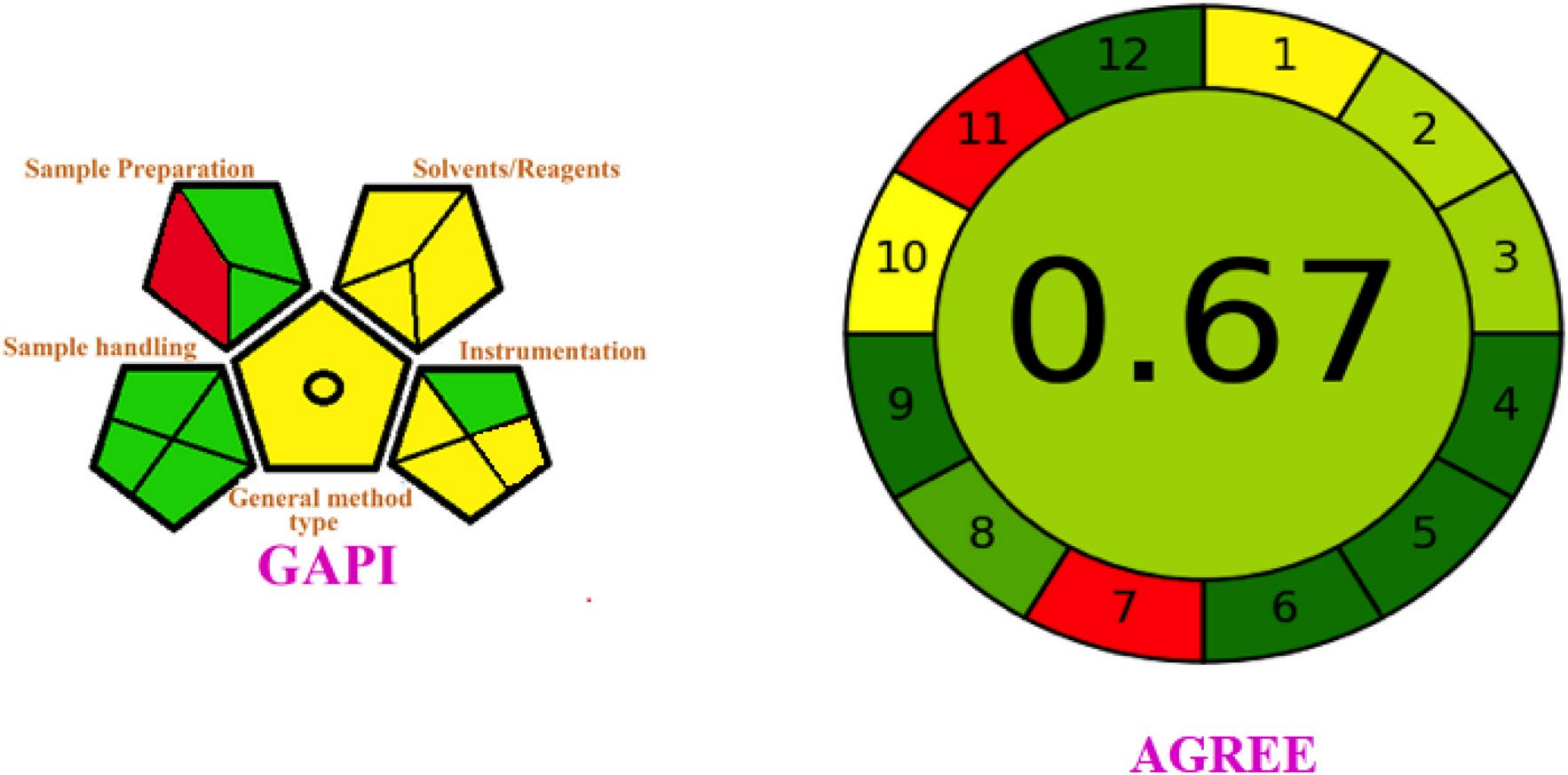
Method greenness assessment tools pictograms.

A clock-shaped pictogram with twelve divisions representing an analytical chemistry principle is called AGREE. A numeric value ranging from 0.0 to 1.0 is represented in the middle of a clock-shaped feature to indicate greenness. Based on the current method used with the AGREE tool, the result was a score of 0.67. The Agree and GAPI figures are shown in Fig. 8. All three principles conclude that the method is green. The GAPI, AGREE, and analytical eco-scale penalty points are tabulated in Supplementary Table 4.

## Conclusion

A simple, rapid, and stability-indicating method is developed based on the AQbD and green chemistry principles with a runtime of 12 min. The protocol is developed using AQbD principles such as risk assessment, screening, and optimization of CMPs using the Design of Experiments. Numerical and graphical optimization were employed while optimizing the CMPs and determining method operable design region conditions. The best suitable diluent was optimized to achieve a stable sample solution. Forced degradation studies were performed on a drug product sample to verify the stability-indicating capability of the method. The acute conditions were identified to take appropriate care during analysis. The MPM was sensitive to basic and acidic conditions. The generated unknown impurities in the base stress study were identified using a Q-ToF MS coupled with UPLC. The contaminants formed are isomeric esters of MPA and sorbitol. The method validation results conclude that the method is suitable, precise, sensitive, linear, specific, stable, and robust for quantifying impurities present in drug substances and oral suspension of MPM. The final method was evaluated for greenness and eco-friendliness using GAPI, AGREE, and analytical eco-scale, and it found that the method is green.

## Supplementary Information

Supplementary Information.

## Data Availability

All data generated or analyzed during this study are included in this published article and its supplementary information files.
