# Supplementary material for: AQbD based green UPLC method to determine mycophenolate mofetil impurities and Identification of degradation products by QToF LCMS
Source: Sci Rep. 2022 Nov 9;12:19138. doi: 10.1038/s41598-022-22998-0 (PMC9646803; doi:10.1038/s41598-022-22998-0)
Supplement: Supplementary file 1 — Supplementary Information. [file 41598_2022_22998_MOESM1_ESM.docx]

**AQbD based green UPLC method to determine mycophenolate mofetil impurities and Identification of degradation products by QToF LCMS**

Siva Krishna Muchakayala^a, b^, Naresh Kumar Katari^b, d*^, Kalyan Kumar Saripella^a^, Henele Schaaf^a^, Vishnu Murthy Marisetti^c^, Leela Prasad Kowtharapu^b^, and Sreekantha Babu Jonnalagadda^d*^

*^a^ Douglas Pharma US Inc, 1035 Louis Drive, Warminster, Pennsylvania, USA-18974.*

*^b^ Department of Chemistry, GITAM School of Science, GITAM deemed to be University, Hyderabad, Telangana, INDIA 502329.* *E-mail:* [*nkatari@gitam.edu*](mailto:nkatari@gitam.edu)*;* [*dr.n.k.katari@gmail.com*](mailto:dr.n.k.katari@gmail.com)

*^c^ Analytical Research and Development, ScieGen pharmaceuticals Inc, 89 Arkay Drive, Hauppauge, New York-11788, USA.*

*^d^School of Chemistry & Physics, College of Agriculture, Engineering & Science, Westville Campus, University of KwaZulu-Natal, P Bag X 54001, Durban-4000, South Africa.* [*Jonnalagaddas@ukzn.ac.za*](mailto:Jonnalagaddas@ukzn.ac.za)

**LIST OF TABLES:**

**Supplementary Table 1** D-optimal split plot screening design data.

**Supplementary Table 2.** Significant model terms from the CCD.

**Supplementary Table 3** Degradation and peak purity results.

**Supplementary Table 4** GAPI, AGREE, and Analytical eco-scale scores.

**LIST OF FIGURES:**

**Supplementary Fig. 1** The chromatogram from the feasibility study. Mobile Phase: Buffer (2 mL TEA in 650 mL; pH 5.3): ACN (700 :1300); Column: Zorbax SB C8, (250 × 4.6)mm, 5 µm; Flow rate 1.5 mL min^-1^; Column oven temperature: 45 °C and Injection Volume: 10 µL.

**Supplementary Fig. 2** A cause-and-effect “Ishikawa” fishbone diagram.

**Supplementary Fig. 3** Numerical optimization plots for the screening D-optimal design

**Supplementary Fig. 4** Numerical optimization plots from the CCD.

**Supplementary Fig. 5** 2D-contour plots of the desirability and the predicted responses.

**Supplementary Fig. 6** The desirability plots from the numerical optimization.

**Supplementary Fig. 7(a)** Positive ion mass chromatogram from the base degradation sample. (a) Unknown at RRT 0.84 (Rt 7.70); (b) Unknown at RRT 0.85 (Rt 7.89); (c) Unknown at RRT 0.87 (Rt 8.05); (d) Mycophenolic acid sorbitol ester (Rt 8.56); (e) MPM (9.60), and (f) MPA (10.76).

**Supplementary Fig. 7(b)** Negative ion mass chromatogram from the base degradation sample. (a) Unknown at RRT 0.84 (Rt 7.70); (b) Unknown at RRT 0.85 (Rt 7.89); (c) Unknown at RRT 0.87 (Rt 8.05); (d) Mycophenolic acid sorbitol ester (Rt 8.55); (e) MPM (9.59), and (f) MPA (10.75).

**Supplementary Fig. 8** Positive mode low energy mass spectrums. (a) Unknown at RRT 0.84; (b) Unknown at RRT 0.85;(c) Unknown at RRT 0.87; (d) Mycophenolic acid sorbitol ester; (e) MPM; and (f) MPA.

**Supplementary Fig. 9** Positive mode high energy mass spectrums. (a) Unknown at RRT 0.84; (b) Unknown at RRT 0.85;(c) Unknown at RRT 0.87; (d) Mycophenolic acid sorbitol ester, (e) MPM, and (f) MPA.

**Supplementary Fig. 10** Negative mode low energy mass spectrums. (a) Unknown at RRT 0.84; (b) Unknown at RRT 0.85;(c) Unknown at RRT 0.87; (d) Mycophenolic acid sorbitol ester, (e) MPM, and (f) MPA.

**Supplementary Fig. 11** Negative mode high energy mass spectrums. a) Unknown at RRT 0.84; (b) Unknown at RRT 0.85;(c) Unknown at RRT 0.87; (d) Mycophenolic acid sorbitol ester, (e) MPM, and (f) MPA.

**Supplementary Table 1** D-optimal split plot screening design.*^a^*

| Run | Factor 1 | Factor 2 | Factor 3 | Factor 4 | Response 1 | Response 2 | Response 3 | Response 4 | Response 5 |
| --- | --- | --- | --- | --- | --- | --- | --- | --- | --- |
|  | A: Mobile Phase | B:Gradient (% MP-A:B) | C: Flow rate  (mL min^-1^) | D: Column Temp. (°C) | R1 | R2 | R3 | R4 | R5 |
| 1 | 25 mM NaH_2_PO_4_ | 60:40 | 0.3 | 30 | 7 | 2.3 | 3.0 | 1.2 | 9.501 |
| 2 | 25 mM NaH_2_PO_4_ | 80:20 | 0.5 | 50 | 8 | 1.7 | 2.5 | 2.7 | 7.905 |
| 3 | 25 mM NaH_2_PO_4_ | 60:40 | 0.5 | 50 | 8 | 1.9 | 2.0 | 2.9 | 6.545 |
| 4 | 25 mM NaH_2_PO_4_ | 80:20 | 0.5 | 30 | 7 | 1.8 | 3.1 | 1.3 | 8.395 |
| 5 | H_2_O | 60:40 | 0.3 | 50 | 7 | 4.8 | 1.0 | 1.8 | 8.738 |
| 6 | H_2_O | 60:40 | 0.5 | 30 | 5 | 3.8 | 2.4 | 1.6 | 7.385 |
| 7 | H_2_O | 80:20 | 0.3 | 30 | 5 | 6.0 | 2.4 | 1.2 | 10.282 |
| 8 | H_2_O | 80:20 | 0.5 | 50 | 5 | 7.6 | 2.6 | 1.2 | 7.933 |
| 9 | H_2_O | 80:20 | 0.3 | 50 | 5 | 10.0 | 1.7 | 1.5 | 9.000 |
| 10 | H_2_O | 60:40 | 0.3 | 30 | 5 | 3.8 | 2.5 | 1.3 | 9.529 |
| 11 | H_2_O | 60:40 | 0.5 | 50 | 7 | 3.8 | 2.3 | 1.8 | 6.586 |
| 12 | H_2_O | 80:20 | 0.5 | 30 | 5 | 6.7 | 2.6 | 1.2 | 8.418 |
| 13 | 25 mM KH_2_PO_4_ | 80:20 | 0.3 | 30 | 7 | 1.9 | 3.0 | 1.2 | 10.282 |
| 14 | 25 mM KH_2_PO_4_ | 60:40 | 0.3 | 50 | 8 | 1.8 | 2.4 | 2.5 | 8.742 |
| 15 | 25 mM KH_2_PO_4_ | 80:20 | 0.5 | 50 | 8 | 1.4 | 2.7 | 2.4 | 7.940 |
| 16 | 25 mM KH_2_PO_4_ | 60:40 | 0.5 | 30 | 8 | 1.9 | 3.5 | 1.3 | 7.370 |
| 17 | 25 mM CH_3_COONH_4_ | 60:40 | 0.3 | 50 | 8 | 1.5 | 2.6 | 2.1 | 8.728 |
| 18 | 25 mM CH_3_COONH_4_ | 80:20 | 0.5 | 50 | 7 | 1.2 | 2.9 | 1.5 | 7.922 |
| 19 | 25 mM CH_3_COONH_4_ | 60:40 | 0.5 | 30 | 7 | 1.6 | 3.0 | 1.1 | 7.350 |
| 20 | 25 mM CH_3_COONH_4_ | 80:20 | 0.3 | 30 | 7 | 1.7 | 3.3 | 1.1 | 10.273 |
| 21 | 25 mM HCOONH_4_ | 80:20 | 0.5 | 30 | 7 | 1.8 | 3.3 | 1.2 | 8.424 |
| 22 | 25 mM HCOONH_4_ | 60:40 | 0.3 | 30 | 7 | 2.3 | 3.5 | 1.1 | 9.548 |
| 23 | 25 mM HCOONH_4_ | 80:20 | 0.3 | 50 | 8 | 1.7 | 2.2 | 2.1 | 9.763 |
| 24 | 25 mM HCOONH_4_ | 60:40 | 0.5 | 50 | 8 | 1.8 | 2.3 | 2.4 | 6.602 |
| 25 | 25 mM NaH_2_PO_4_ | 60:40 | 0.5 | 30 | 7 | 2.0 | 3.3 | 1.4 | 7.343 |
| 26 | 25 mM NaH_2_PO_4_ | 80:20 | 0.3 | 30 | 7 | 2.1 | 2.8 | 1.3 | 10.249 |
| 27 | 25 mM NaH_2_PO_4_ | 80:20 | 0.3 | 50 | 8 | 1.8 | 2.0 | 2.5 | 9.720 |
| 28 | 25 mM NaH_2_PO_4_ | 60:40 | 0.3 | 50 | 8 | 2.0 | 2.1 | 2.7 | 8.707 |
| 29 | 0.1% HCOOH | 80:20 | 0.5 | 50 | 7 | 1.0 | 2.6 | 2.1 | 10.341 |
| 30 | 0.1% HCOOH | 60:40 | 0.3 | 50 | 7 | 1.3 | 2.4 | 2.1 | 8.722 |
| 31 | 0.1% HCOOH | 60:40 | 0.5 | 30 | 6 | 1.4 | 3.5 | 1.1 | 7.362 |
| 32 | 0.1% HCOOH | 80:20 | 0.3 | 30 | 7 | 1.5 | 2.9 | 1.1 | 10.280 |

*^a^* R1: Number of impurity peaks separated with a minimum resolution of 1.5; R2: Rs between Imp-H and MPM; R3: Rs between MPM and Imp-D; R4: Rs between Imp-D and Imp-F; and R5: Rt of the late eluting peak.

**Supplementary Table 2** Significant model terms from the CCD.*^a^*

| Source | p-Value for the Responses | | | | |
| --- | --- | --- | --- | --- | --- |
|  | R1 | R2 | R3 | R4 | R5 |
| **Model** | < 0.0001 | < 0.0001 | 0.0304 | 0.0108 | 0.0010 |
| A-pH of the Buffer | < 0.0001 | < 0.0001 | 0.0030 | 0.0004 | < 0.0001 |
| B-Initial Gradient | 0.3302 | 0.2386 | 0.8304 | 0.3436 | 0.6585 |
| C-Flow Rate | 0.0339 | 0.0060 | 0.1644 | 0.2133 | 0.9576 |
| D-Column Temp | < 0.0001 | 0.0104 | 0.0208 | 0.0178 | 0.1845 |
| AB | 0.6067 | 0.2193 | 0.7932 | 0.2212 | 0.6798 |
| AC | 0.8248 | 0.0140 | 0.0707 | 0.2097 | 0.6261 |
| AD | 0.1820 | 0.7534 | 0.8750 | 0.2212 | 0.0226 |
| BC | 0.4729 | 0.3519 | 0.6134 | 0.3477 | 0.5495 |
| BD | 0.9177 | 0.2334 | 0.7401 | 0.3995 | 0.7354 |
| CD | 0.9647 | 0.0129 | 0.0916 | 0.5175 | 0.3536 |

*^a^* R1: Rs between Imp-E & A; R2: Rs between Imp-D & MPM; R3: Rs between MPM & Imp-F; R4: Rs between Imp-F & B; R5: Rs between Imp-B & C.

**Supplementary Table 3** Degradation and peak purity results

| Sample Name/ Degradation Type | %Impurities | Purity Angle | Purity Threshold | Purity Flag |
| --- | --- | --- | --- | --- |
| Standard | NA | 0.676 | 1.473 | No |
| As such sample | 0.5746 | 0.035 | 1.892 | No |
| Acid (1.0 N HCl/ 1 mL/ 24 h @ RT) | 6.7276 | 0.091 | 1.841 | No |
| Base (1.0 N NaOH/ 1 mL/24 h @ RT) | 31.1530 | 0.025 | 1.747 | No |
| Peroxide (6% H_2_O_2_/ 1 mL/ 24 h @ RT) | 0.9313 | 0.041 | 1.932 | No |
| Thermal (24 h @ 70°C) | 1.9691 | 0.029 | 1.553 | No |
| Photolytic (1200 KLX/ 200-Watt h m^-2^) | 0.5349 | 0.037 | 1.895 | No |

**Supplementary Table 4** GAPI, AGREE, and Analytical eco-scale scores.

| S.No | Assessment tool | Principle/ Reagents/Chemicals | Score /Penalty points |
| --- | --- | --- | --- |
| 1 | GAPI | 1. Sample collection |  |
|  |  | 1. Sample preservation |  |
|  |  | 1. Sample transport |  |
|  |  | 1. Sample storage |  |
|  |  | 1. Type of method |  |
|  |  | 1. Scale of extraction |  |
|  |  | 1. Solvents/reagents used |  |
|  |  | 1. additional treatments |  |
|  |  | 1. Amount of reagents or solvents |  |
|  |  | 1. Health Hazard |  |
|  |  | 1. Safety hazard |  |
|  |  | 1. Energy per sample |  |
|  |  | 1. Occupational hazard |  |
|  |  | 1. Waste |  |
|  |  | 1. Waste treatment |  |
| 2 | AGREE | 1. Analysis technique – Off-line | 0.48 |
|  |  | 1. Sample size – 1.2 mL | 0.62 |
|  |  | 1. Measurement type – On-line | 0.66 |
|  |  | 1. Analysis processes – 3 or fewer | 1.0 |
|  |  | 1. Automation and miniaturization – Yes | 1.0 |
|  |  | 1. Derivatization – None | 1.0 |
|  |  | 1. Waste – 200 mL | 0.0 |
|  |  | 1. Number of analytes – 9 and 4 | 0.82 |
|  |  | 1. Energy consumption – UPLC | 1.0 |
|  |  | 1. Renewable reagents – Some | 0.5 |
|  |  | 1. Toxic reagents – 45 mL | 0.01 |
|  |  | 1. Operator safety – None | 1.0 |
|  |  | Method AGREE Score | 0.67 |
| 3 | Analytical eco-scale | 1. Phosphate buffer | 0 |
|  |  | 1. Acetonitrile | 4 |
|  |  | 1. Orthophosphoric acid | 4 |
|  |  | 1. HPLC | 1 |
|  |  | 1. Occupational hazard | 0 |
|  |  | 1. Sonicator | 1 |
|  |  | 1. Waste | 5 |
|  |  | 1. Total penalty points | 15 |
|  |  | 1. Analytical Eco-Scale | 85 |


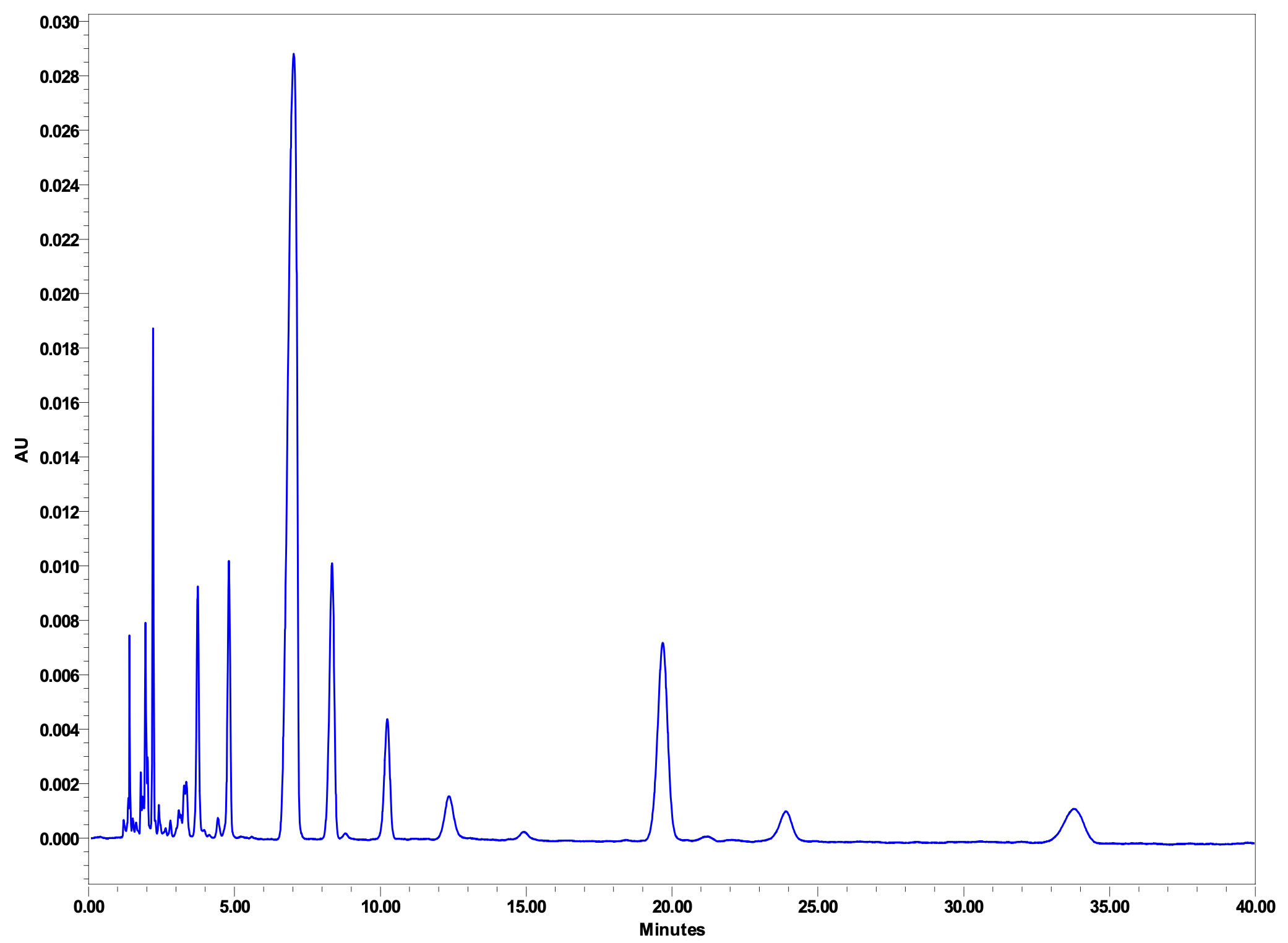


**Supplementary Fig. 1** The chromatogram from the feasibility study. Mobile Phase: Buffer (2 mL TEA in 650 mL; pH 5.3): ACN (700 :1300); Column: Zorbax SB C8, (250 × 4.6)mm, 5 µm; Flow rate 1.5 mL min^-1^; Column oven temperature: 45 °C and Injection Volume: 10 µL.

**Supplementary Fig. 2** A cause-and-effect “Ishikawa” fishbone diagram.


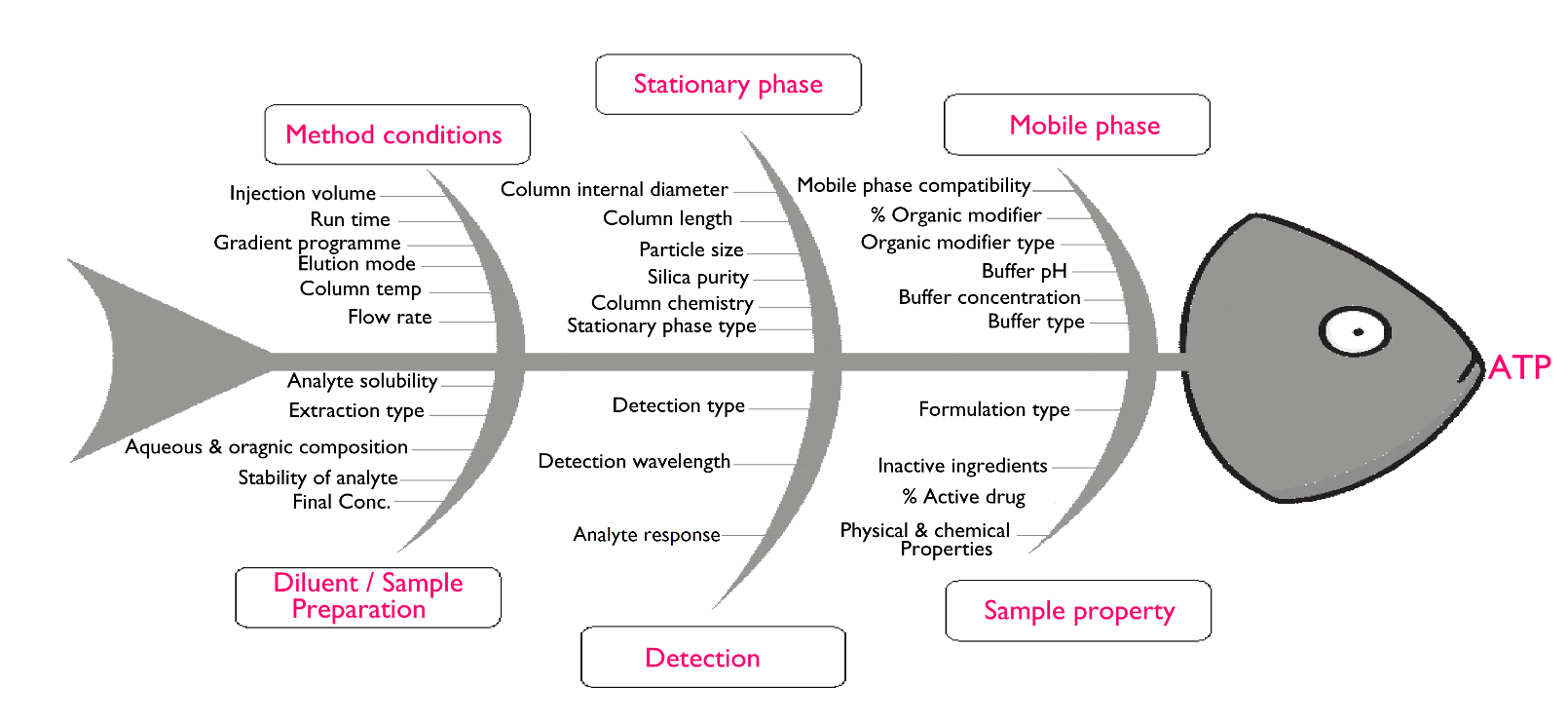


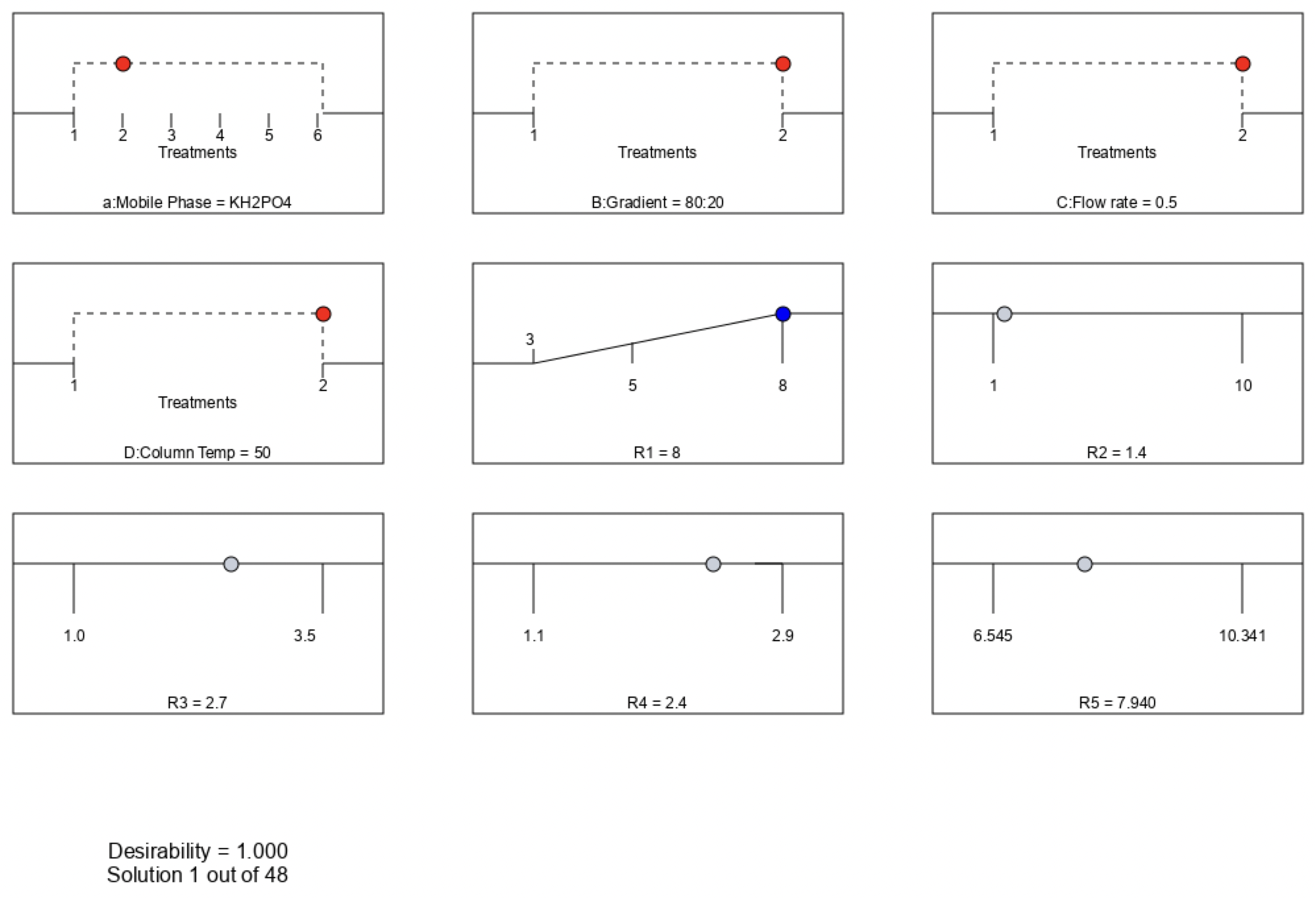


**Supplementary Fig. 3**  Numerical optimization plots from the screening D-optimal design.


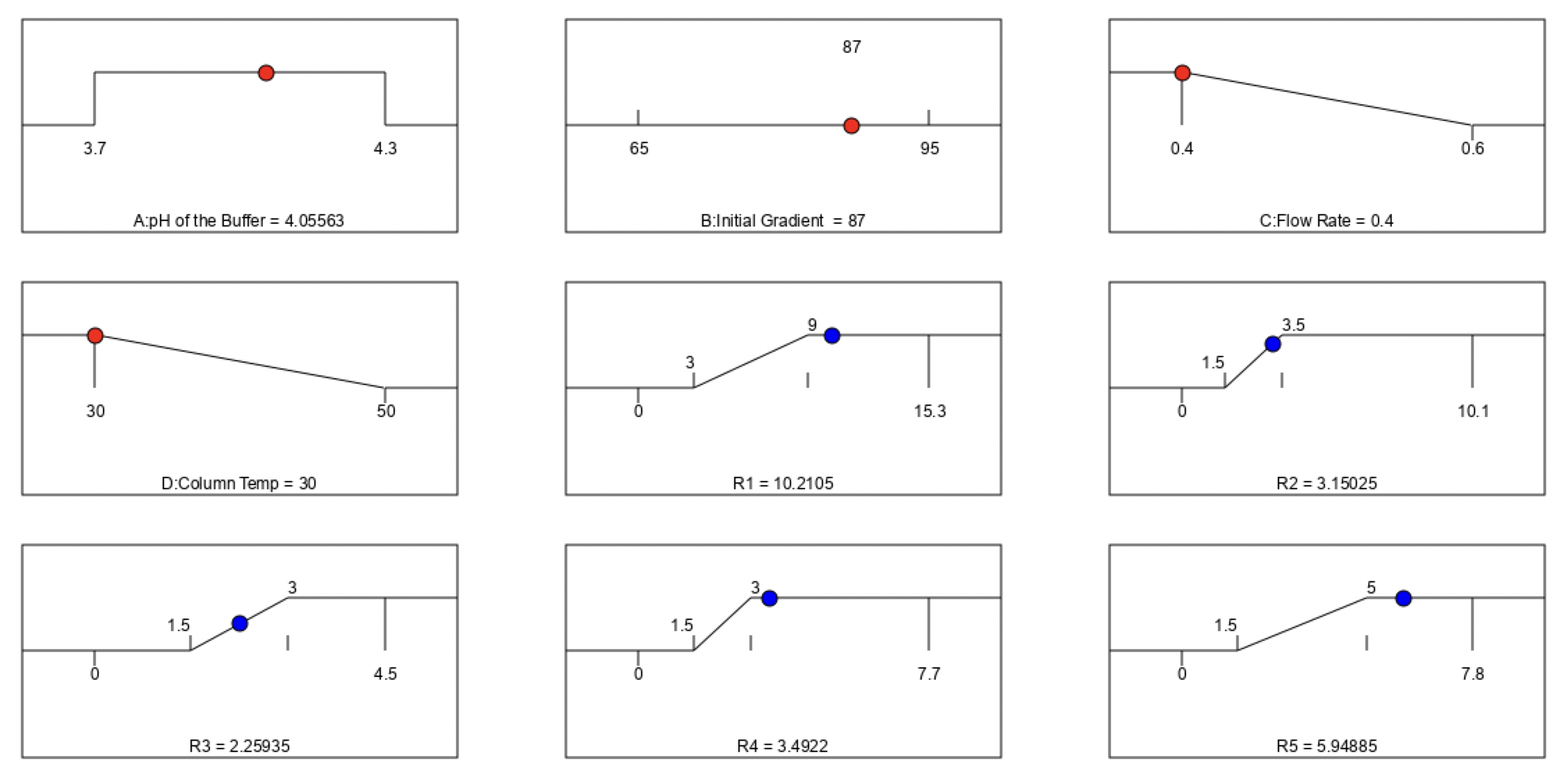


**Supplementary Fig. 4** Numerical optimization plots from the CCD.


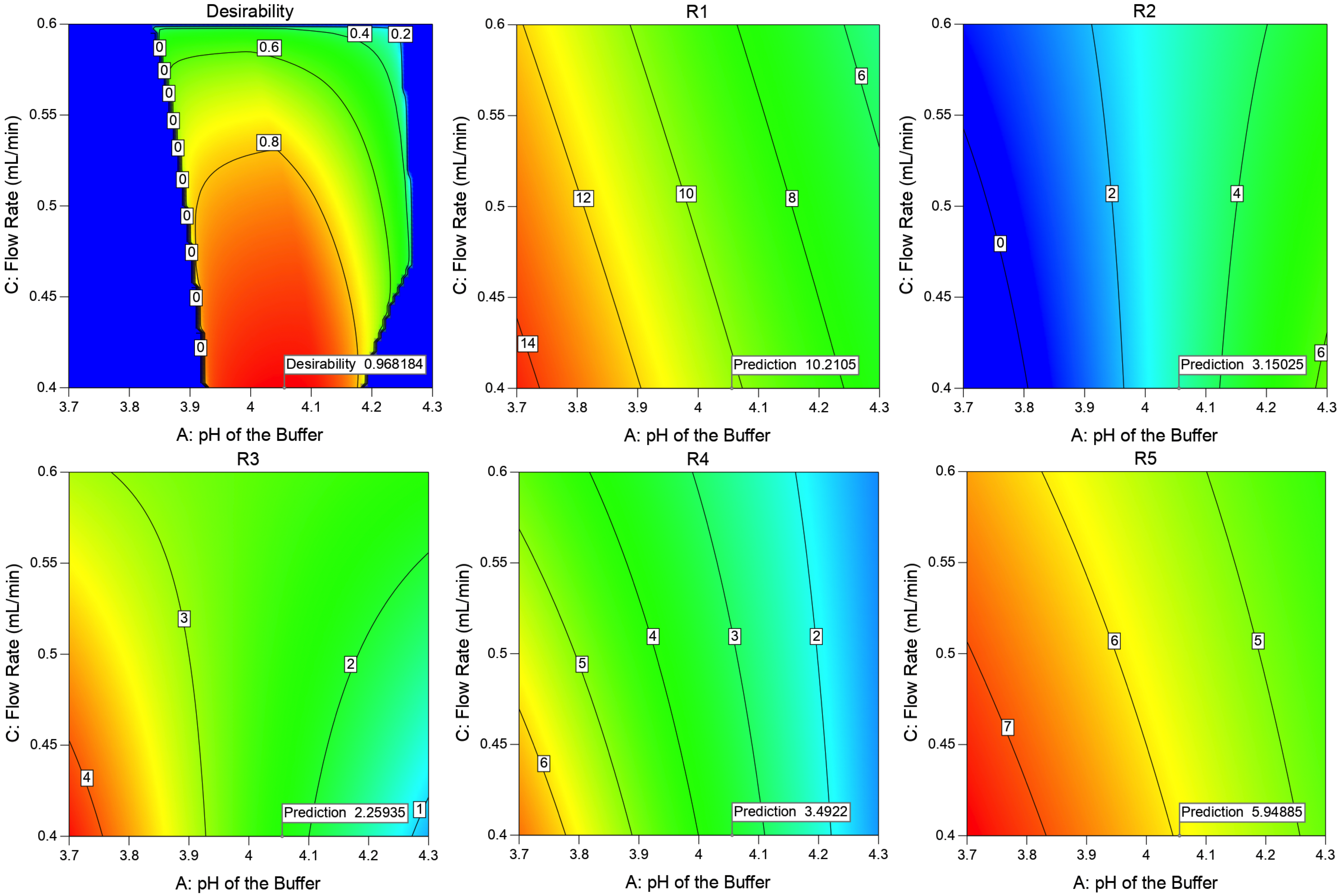


**Supplementary Fig. 5** 2D-contour plots of the desirability and the predicted responses.

**
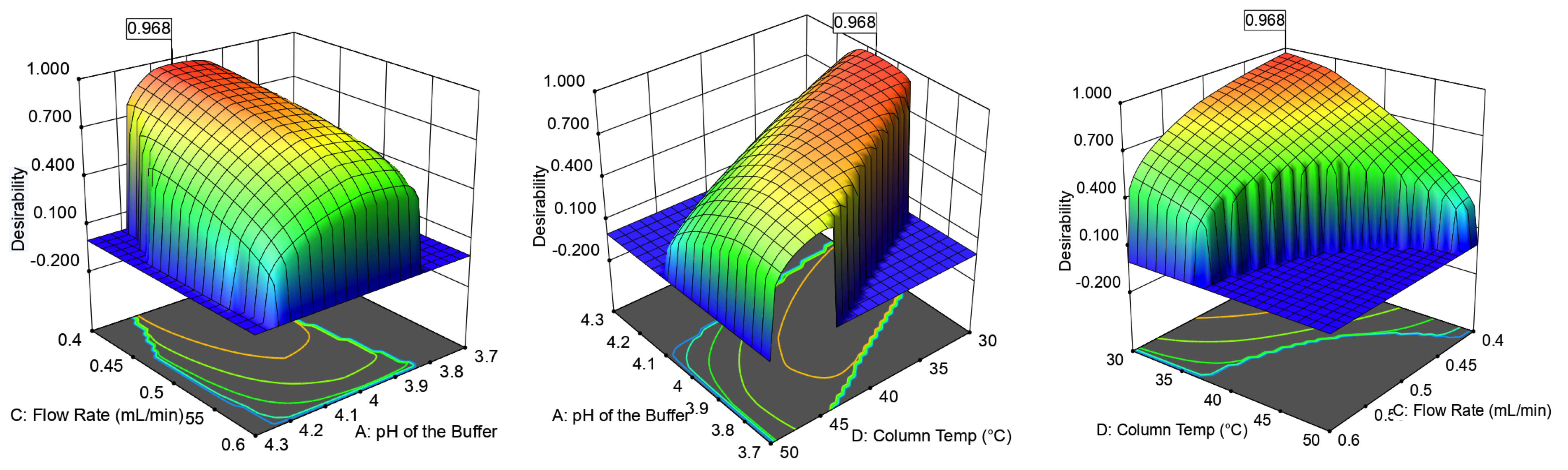
**

**Supplementary Fig. 6** The desirability plots from the numerical optimization.


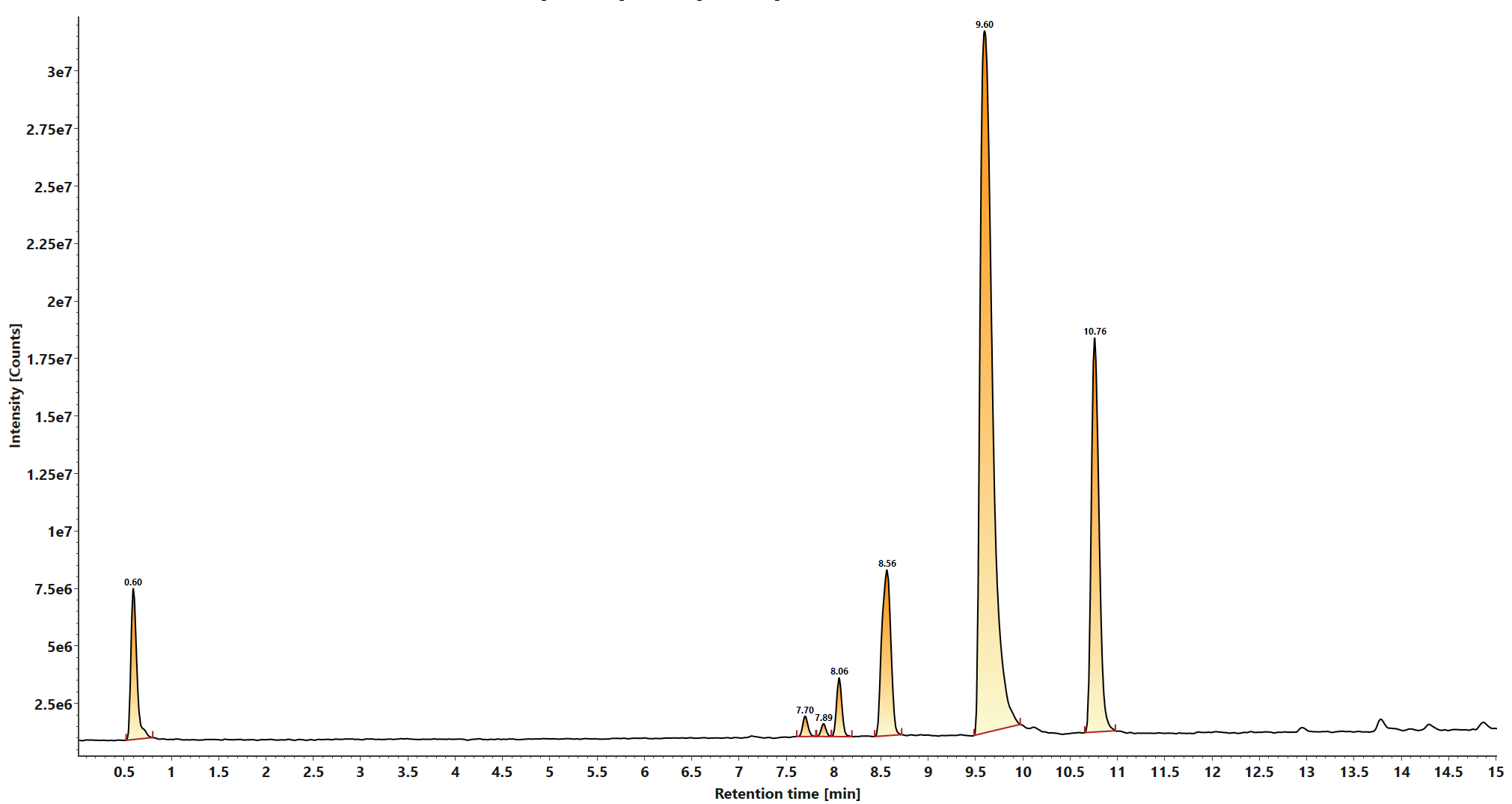


**Supplementary Fig. 7(a)**Positive ion mass chromatogram from the base degradation sample. (a) Unknown at RRT 0.84 (Rt 7.70); (b) Unknown at RRT 0.85 (Rt 7.89); (c) Unknown at RRT 0.87 (Rt 8.05); (d) Mycophenolic acid sorbitol ester (Rt 8.56); (e) MPM (9.60), and (f) MPA (10.76).


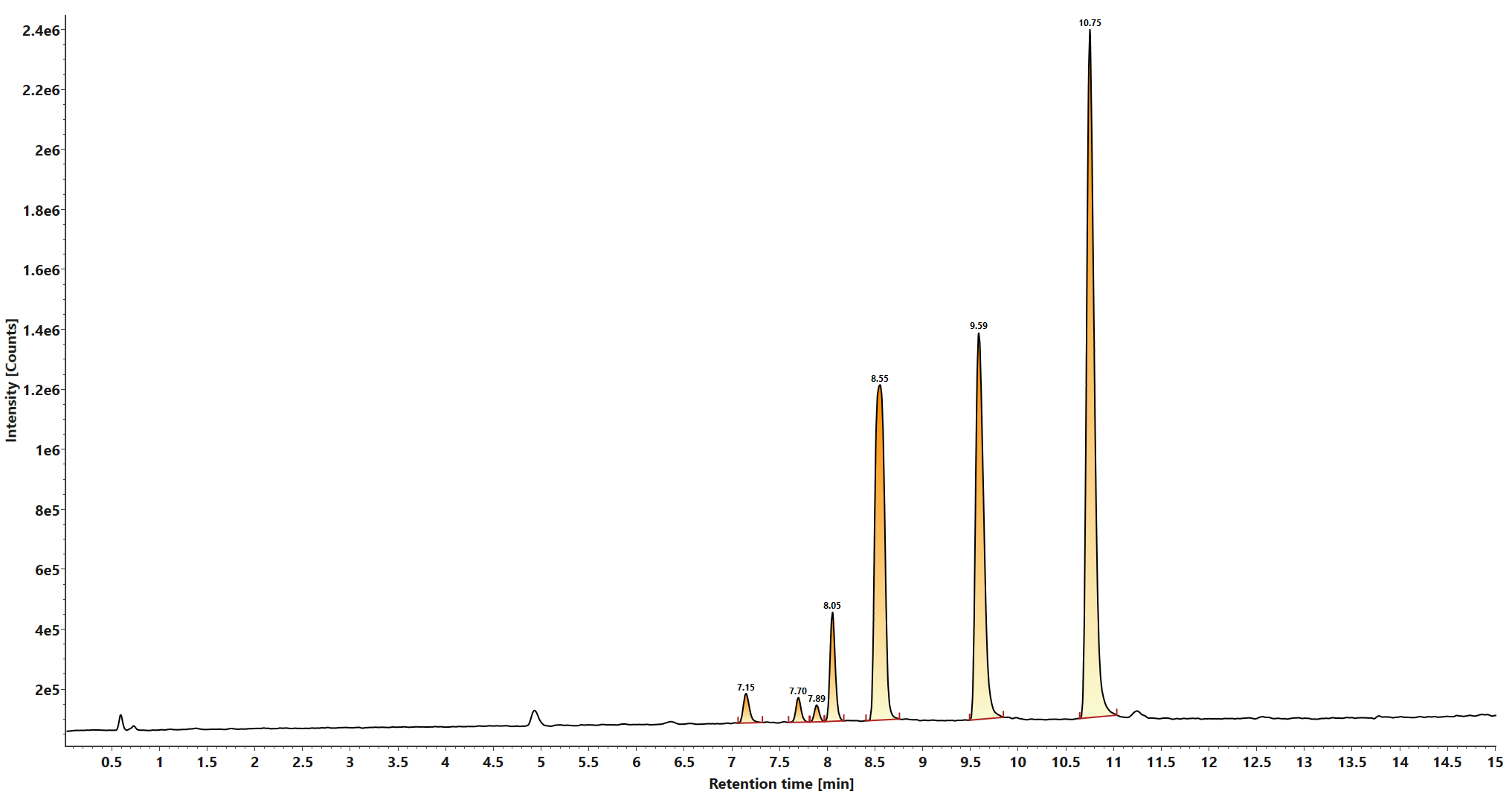


**Supplementary Fig. 7(b)** Negative ion mass chromatogram from the base degradation sample. (a) Unknown at RRT 0.84 (Rt 7.70); (b) Unknown at RRT 0.85 (Rt 7.89); (c) Unknown at RRT 0.87 (Rt 8.05); (d) Mycophenolic acid sorbitol ester (Rt 8.55); (e) MPM (9.59), and (f) MPA (10.75).


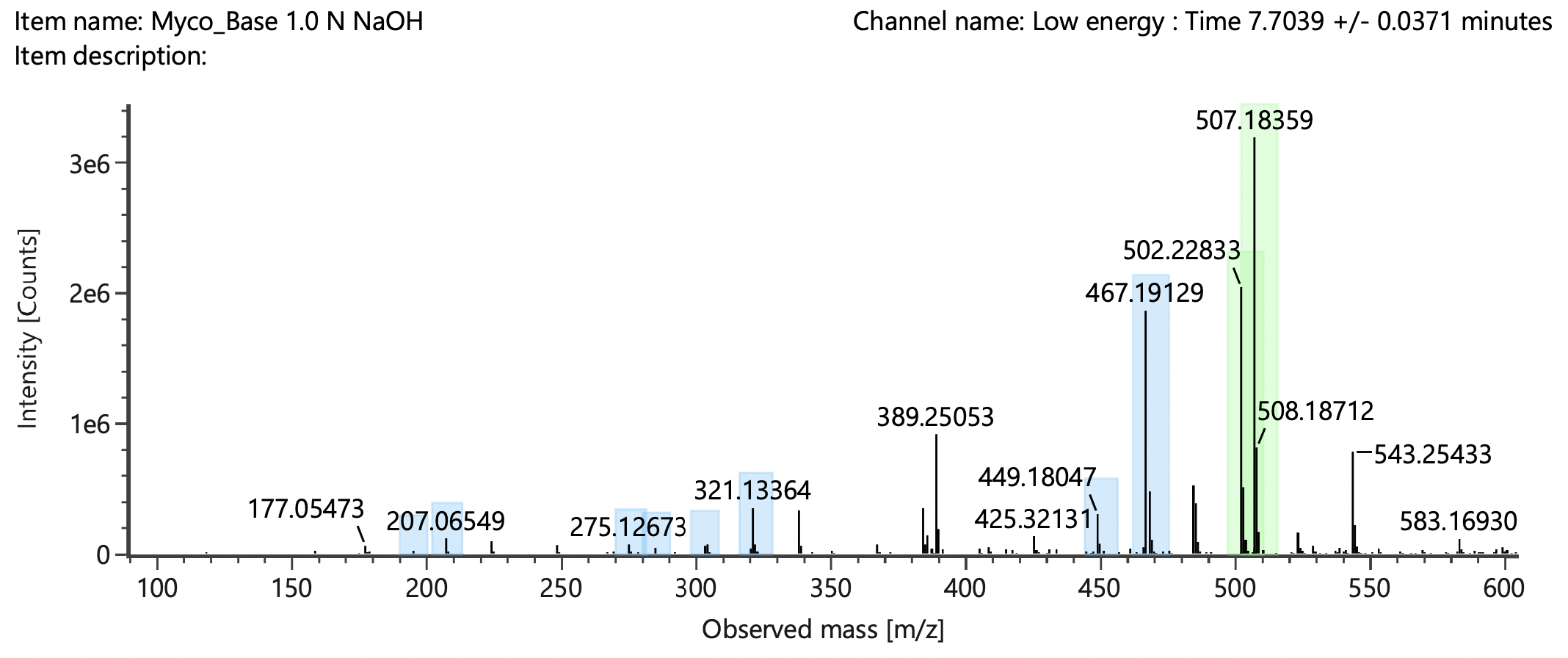


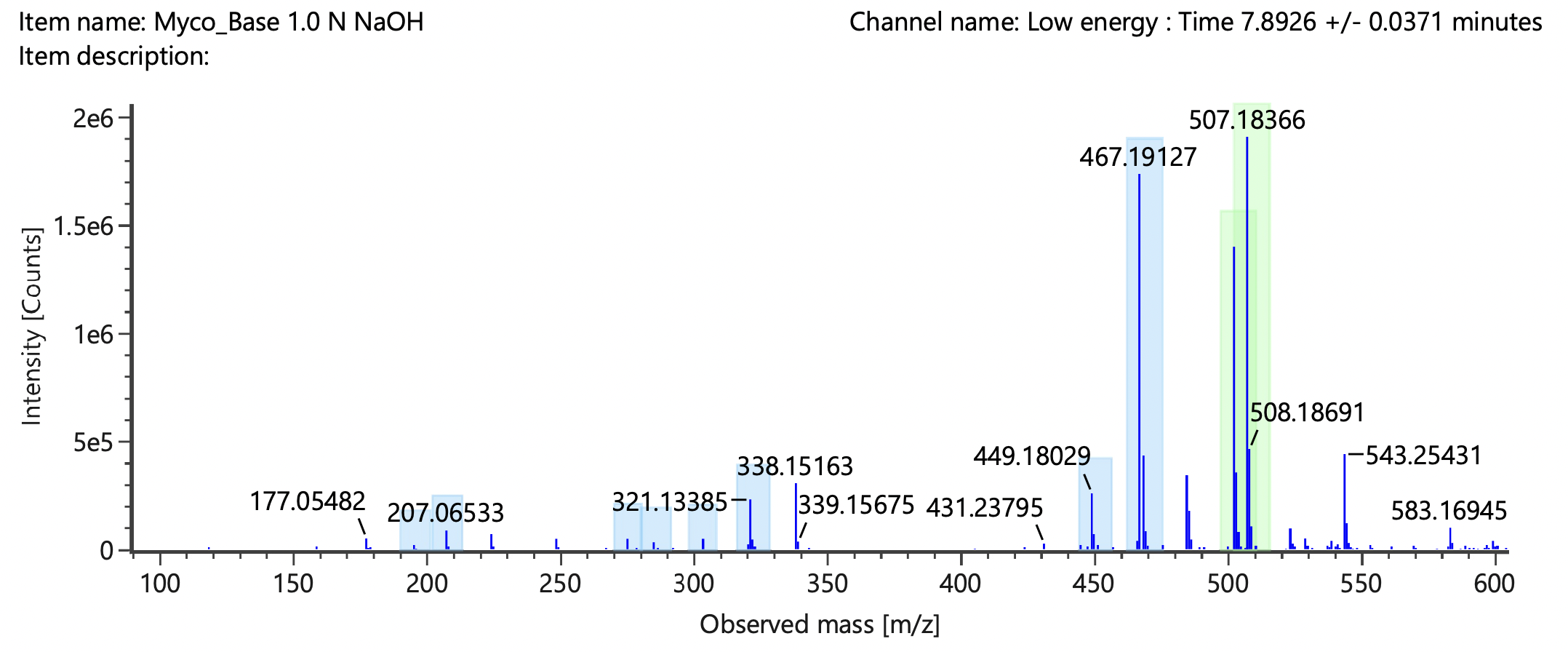


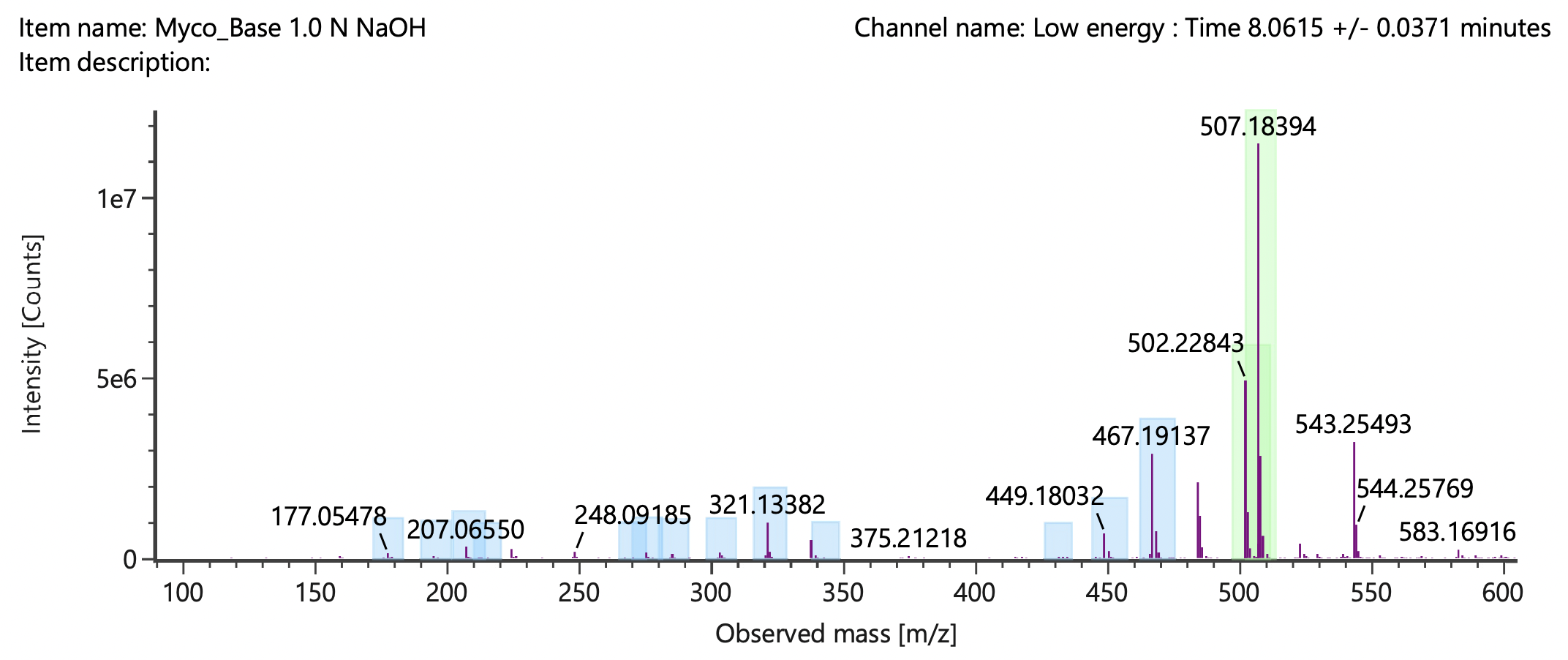


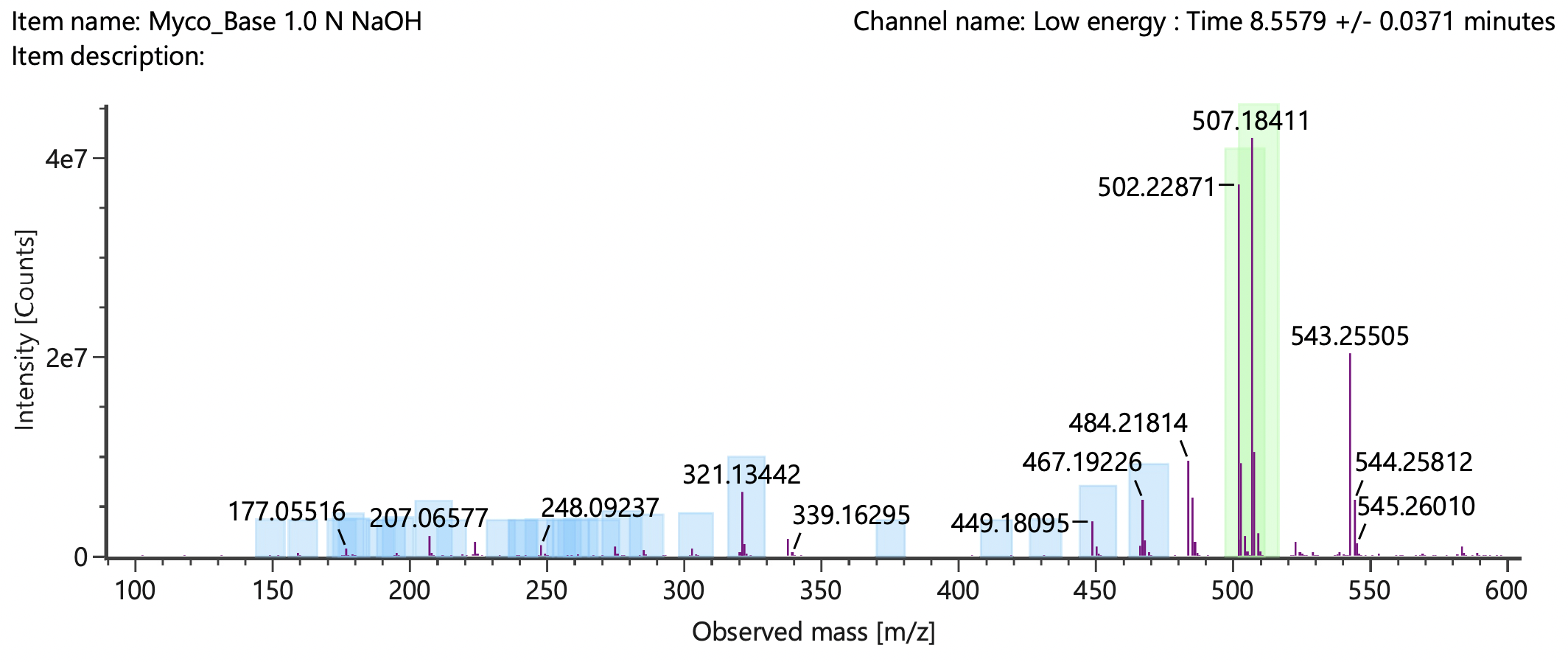


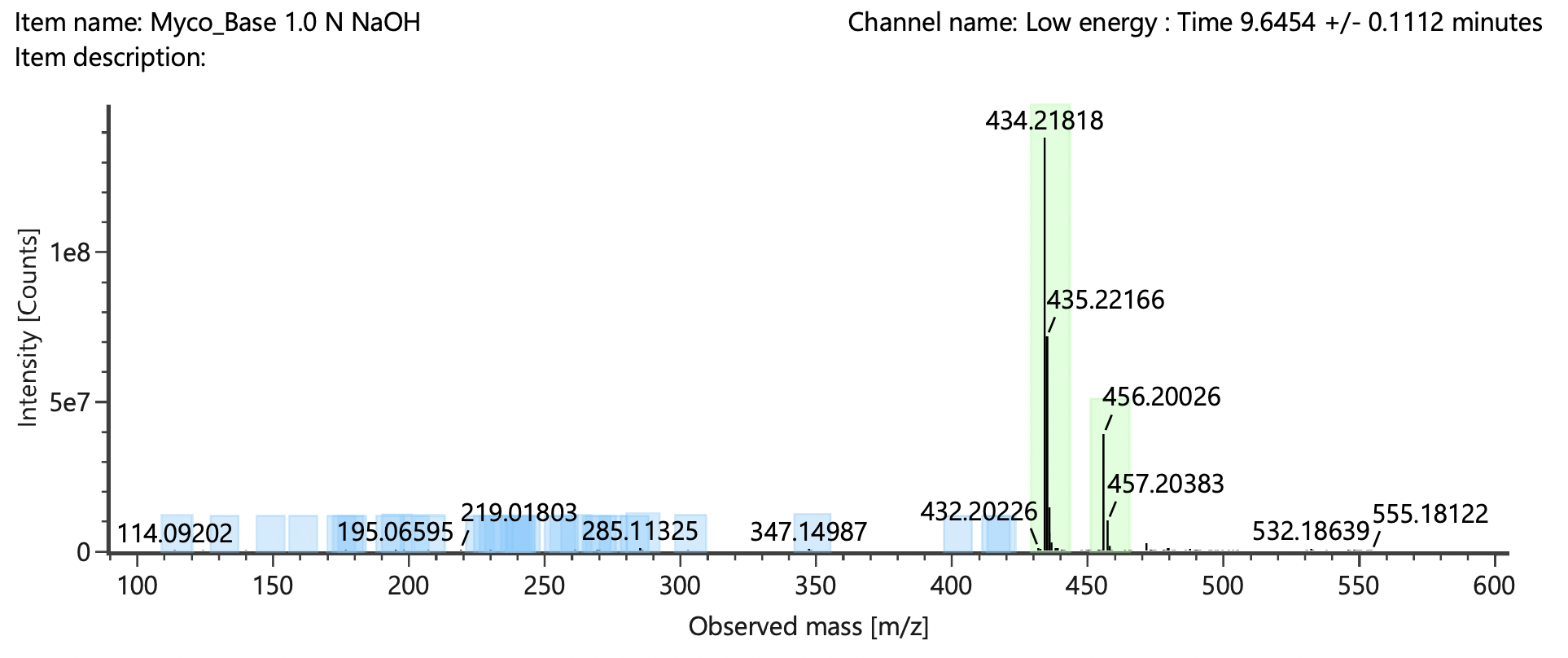


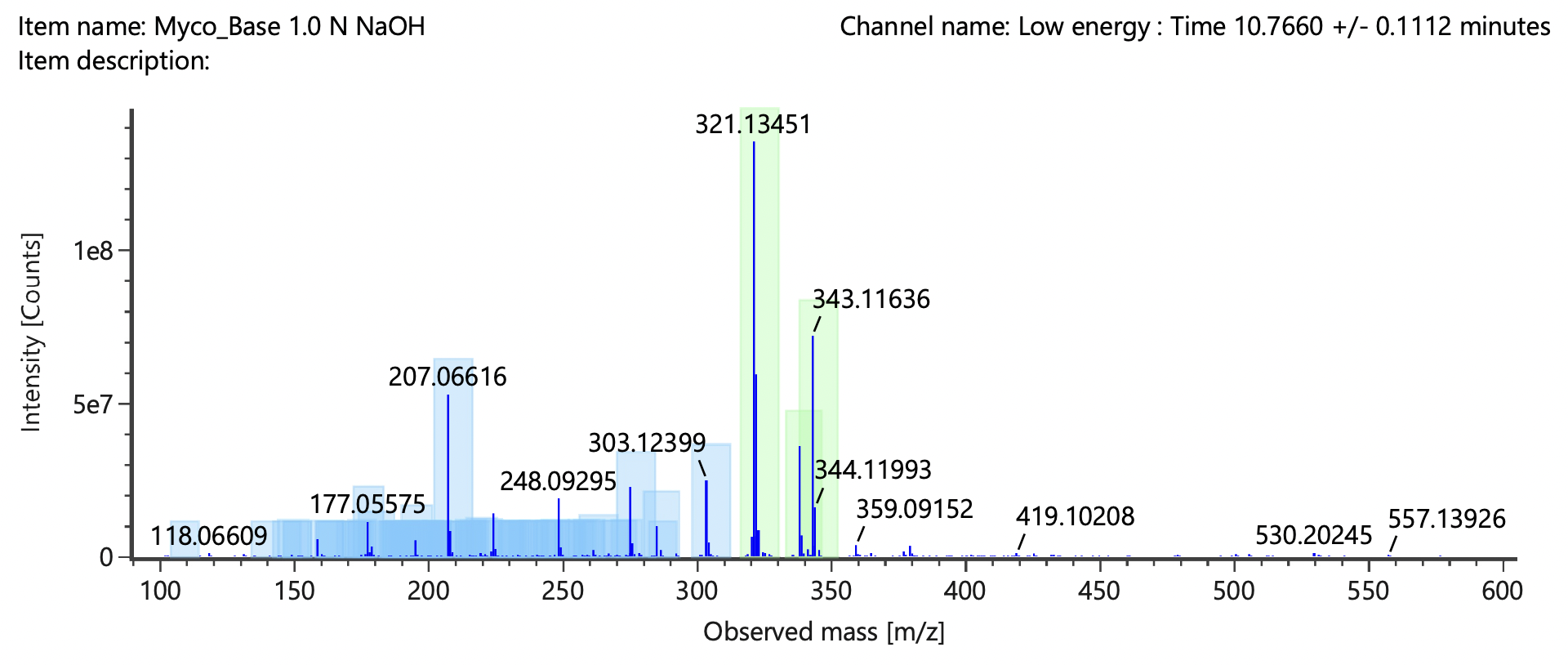


**Supplementary Fig. 8** Positive mode low energy mass spectrums. (a) Unknown at RRT 0.84; (b) Unknown at RRT 0.85;(c) Unknown at RRT 0.87; (d) Mycophenolic acid sorbitol ester; (e) MPM; and (f) MPA.


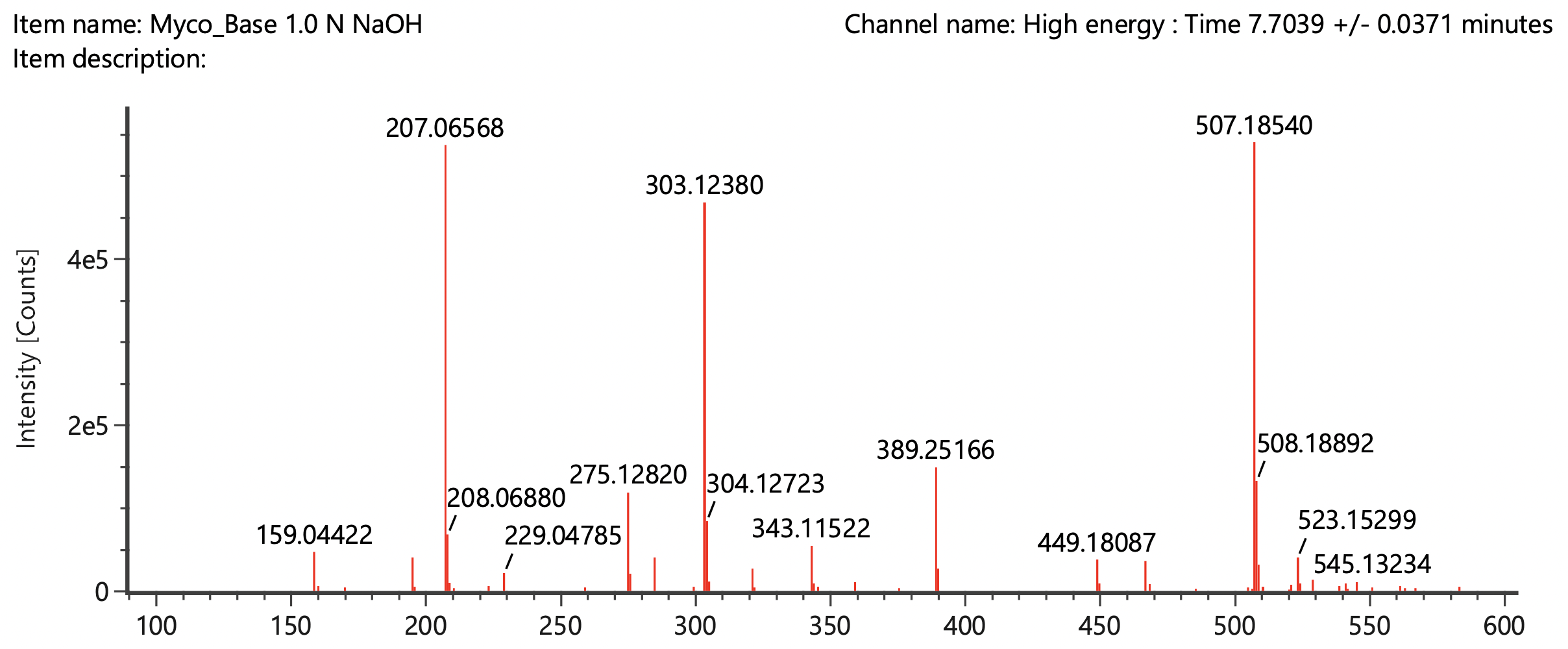


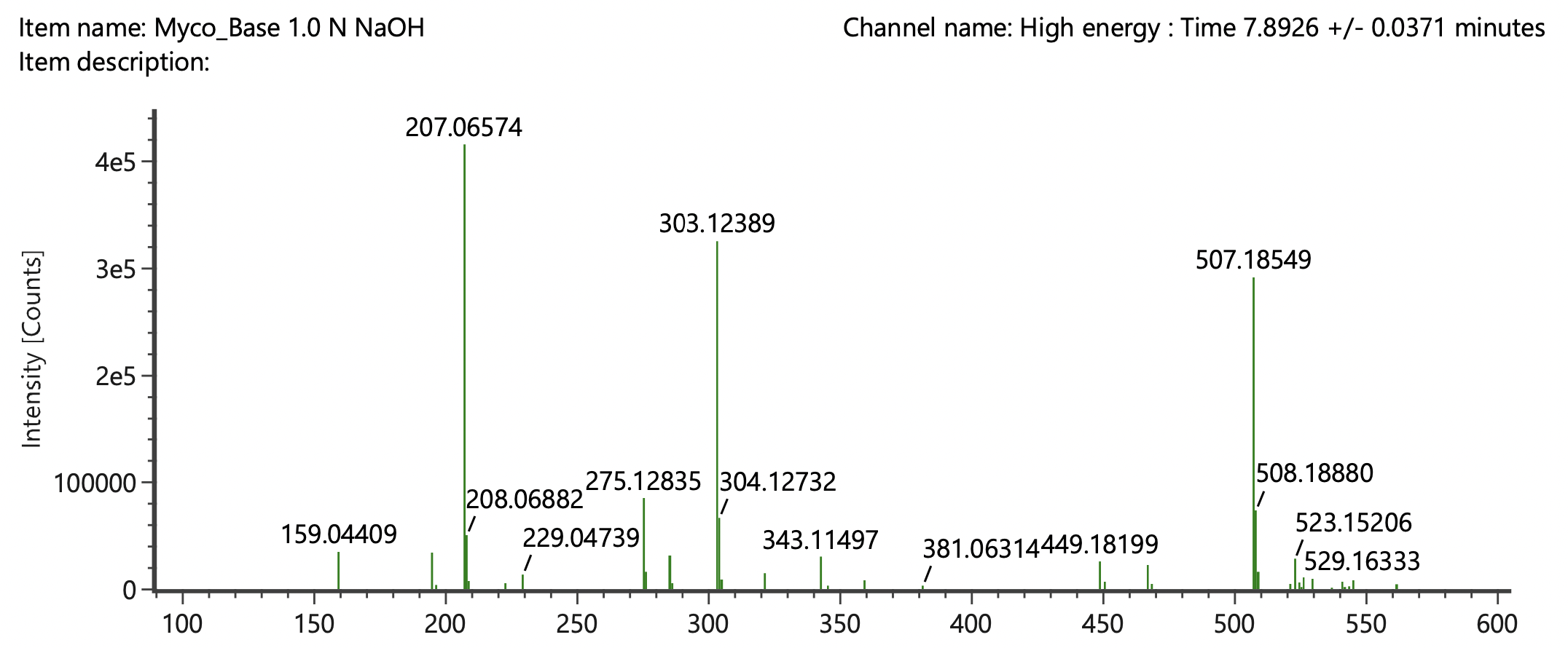


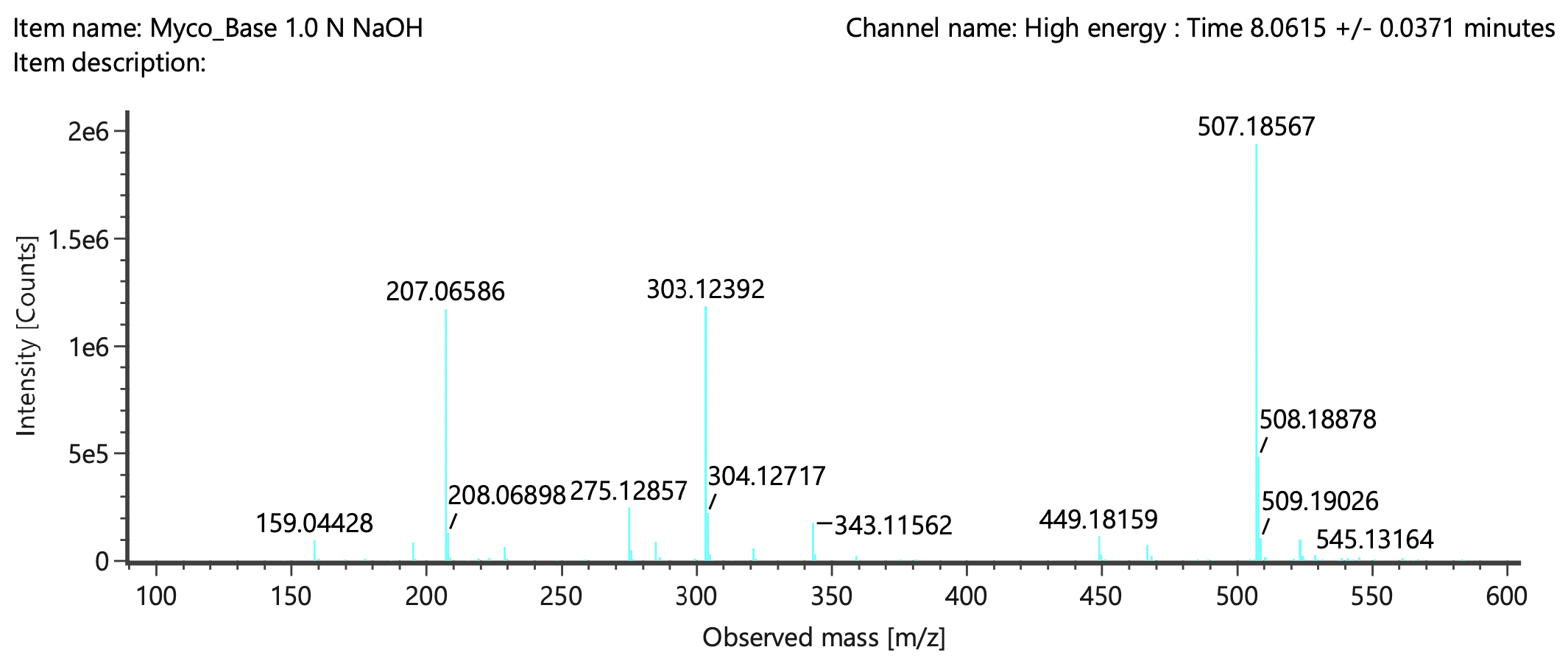


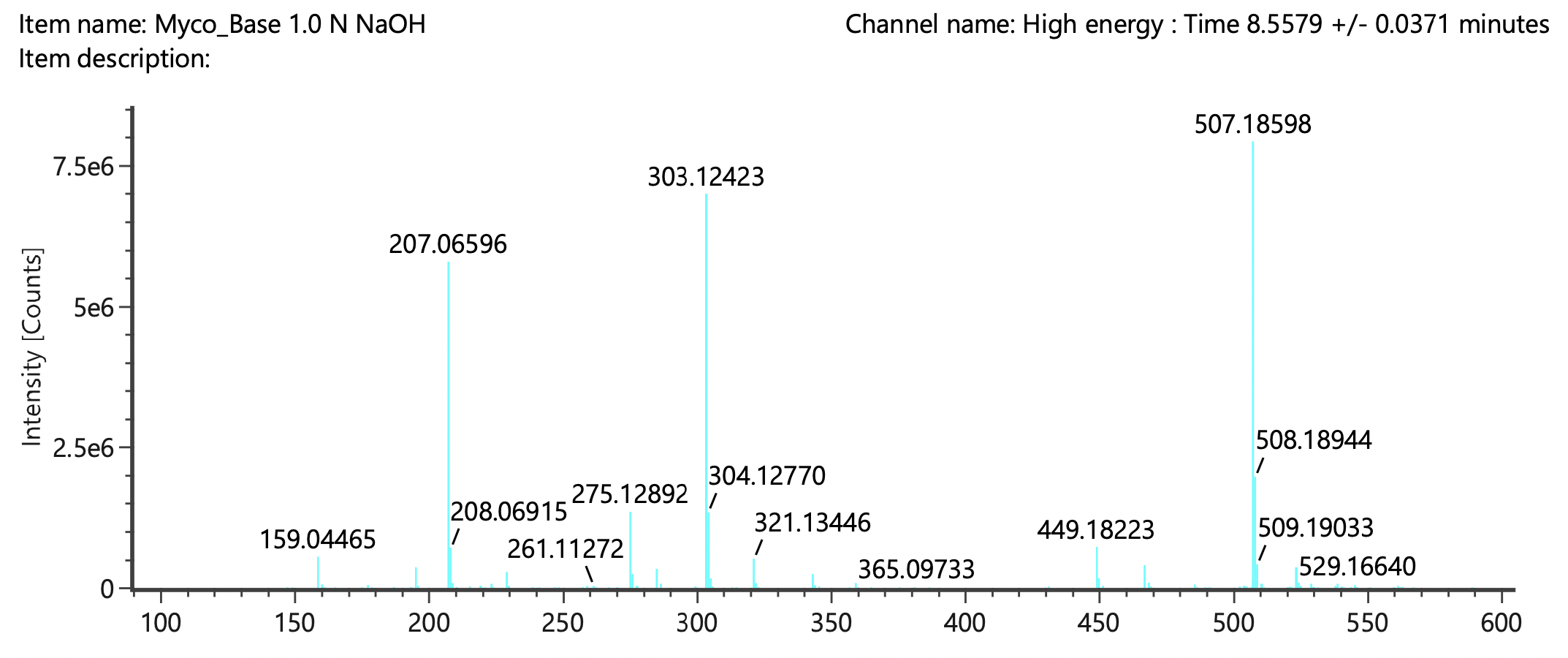


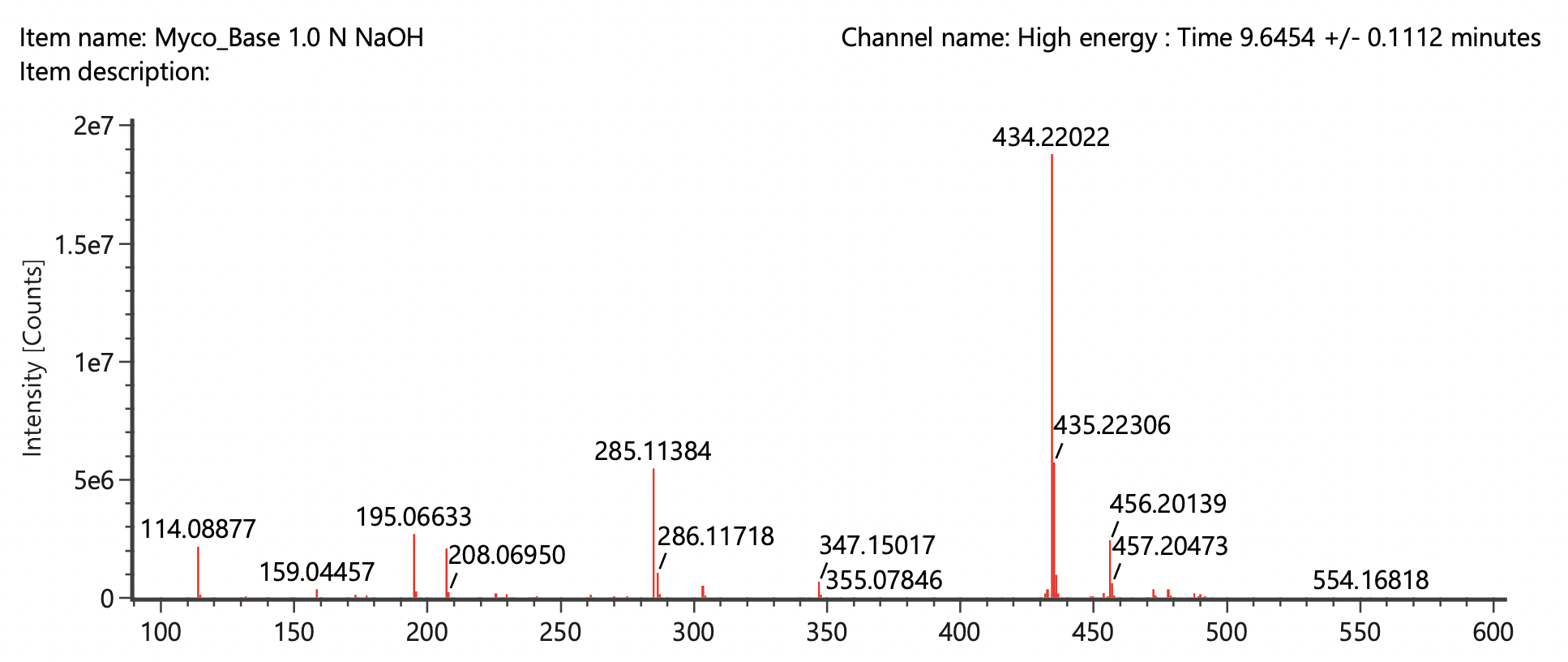


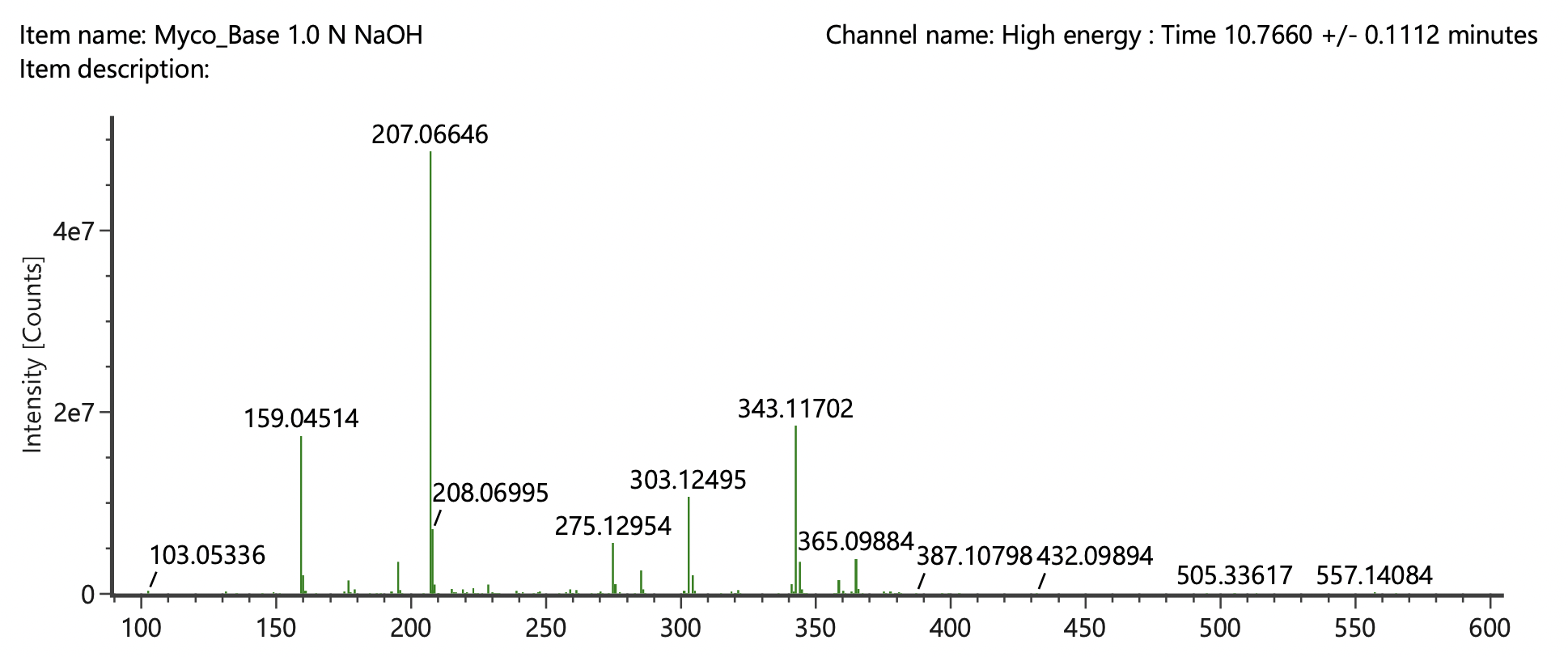


**Supplementary Fig. 9** Positive mode high energy mass spectrums. (a) Unknown at RRT 0.84; (b) Unknown at RRT 0.85;(c) Unknown at RRT 0.87; (d) Mycophenolic acid sorbitol ester, (e) MPM, and (f) MPA.


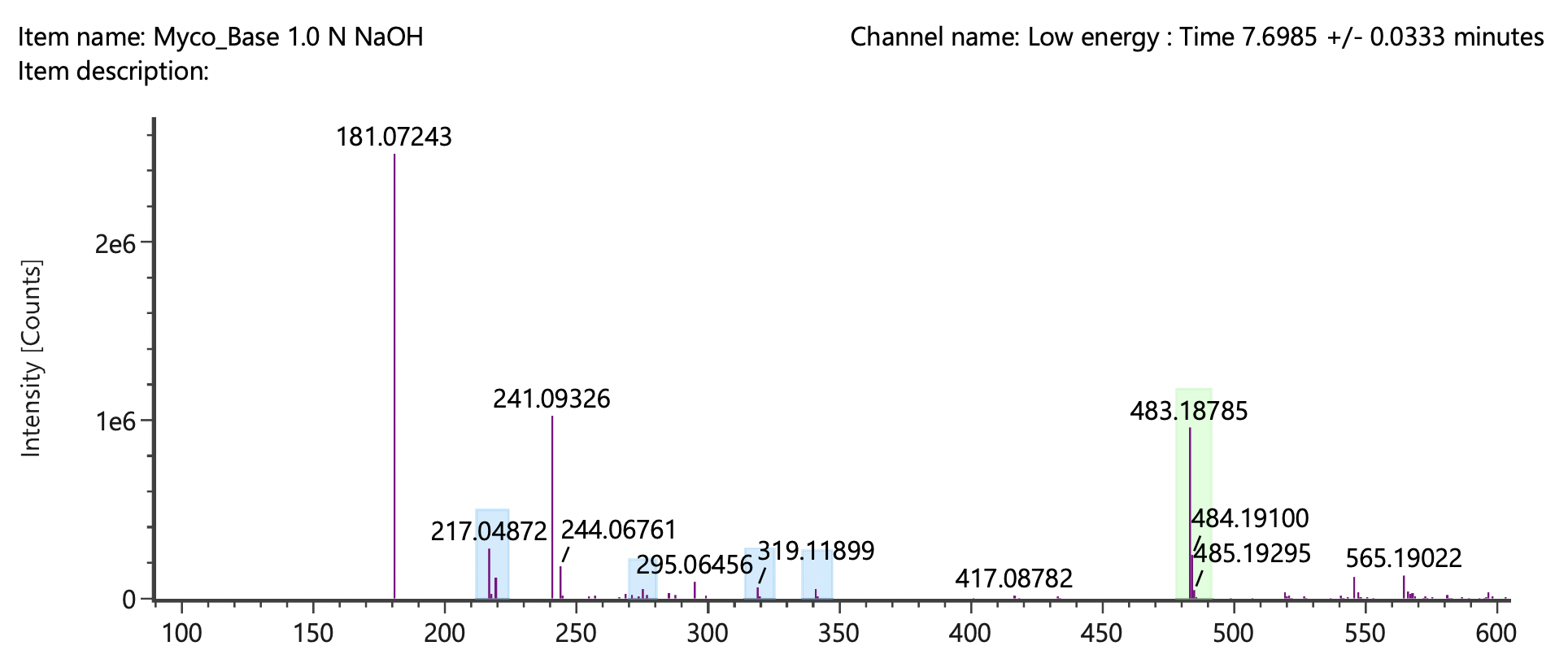


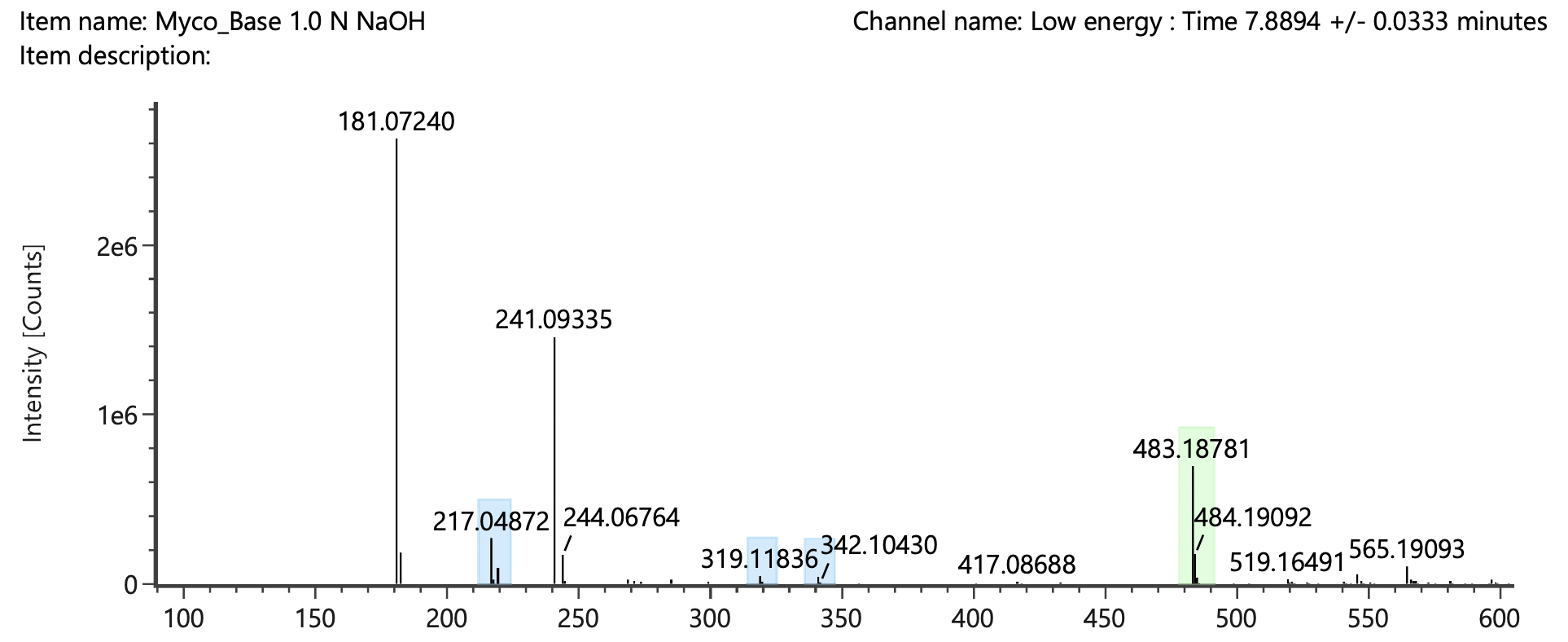


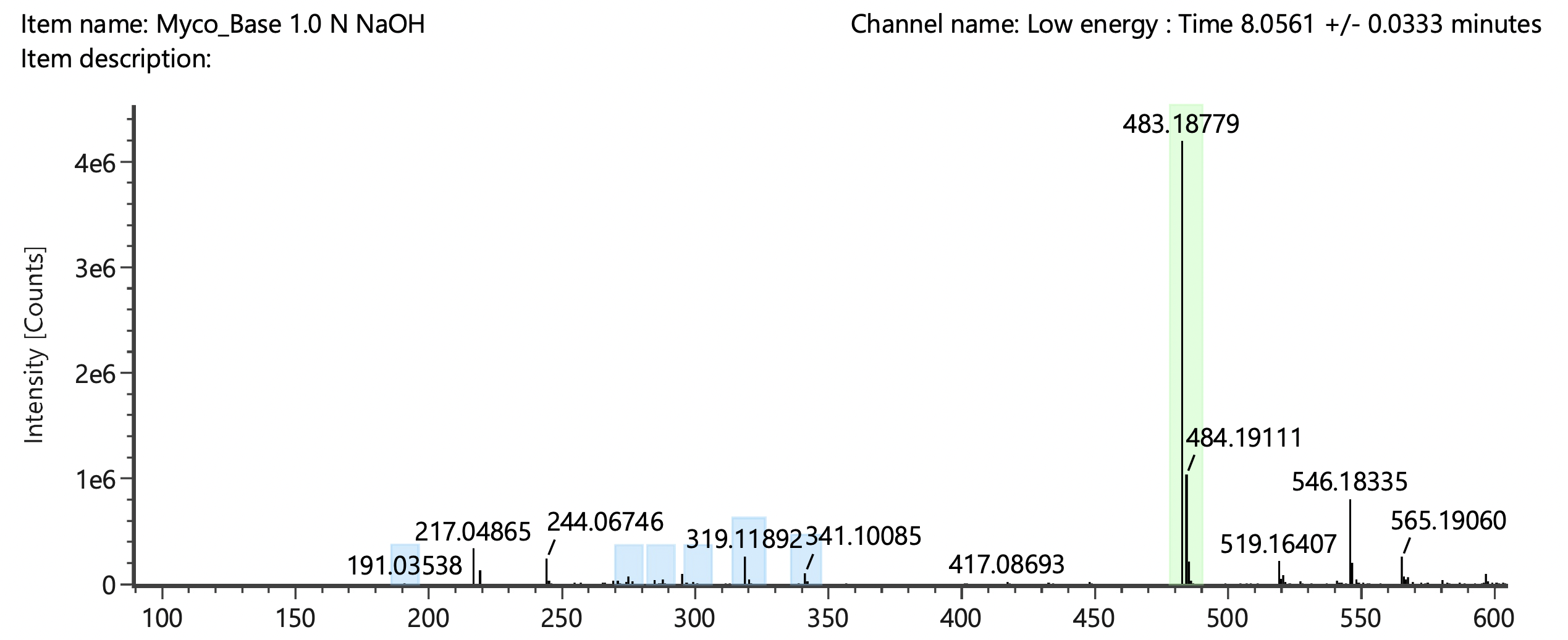


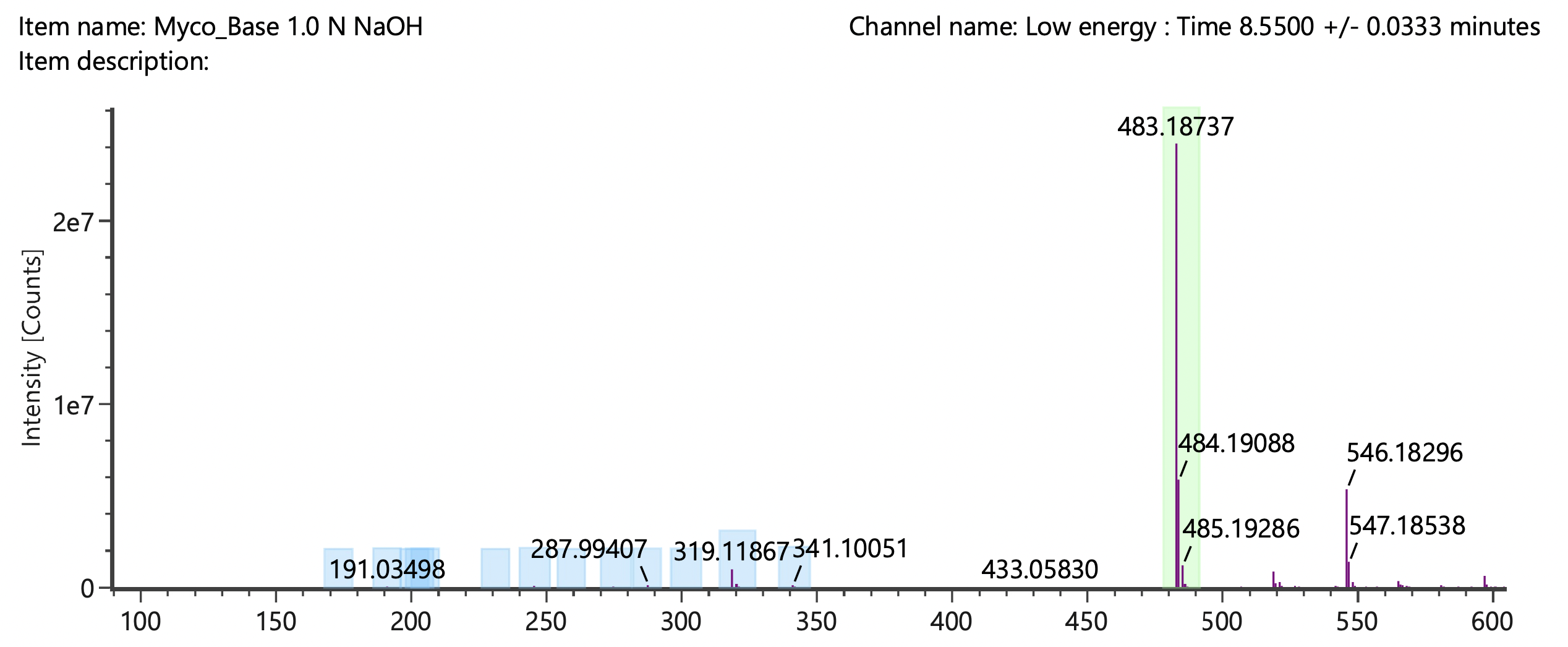


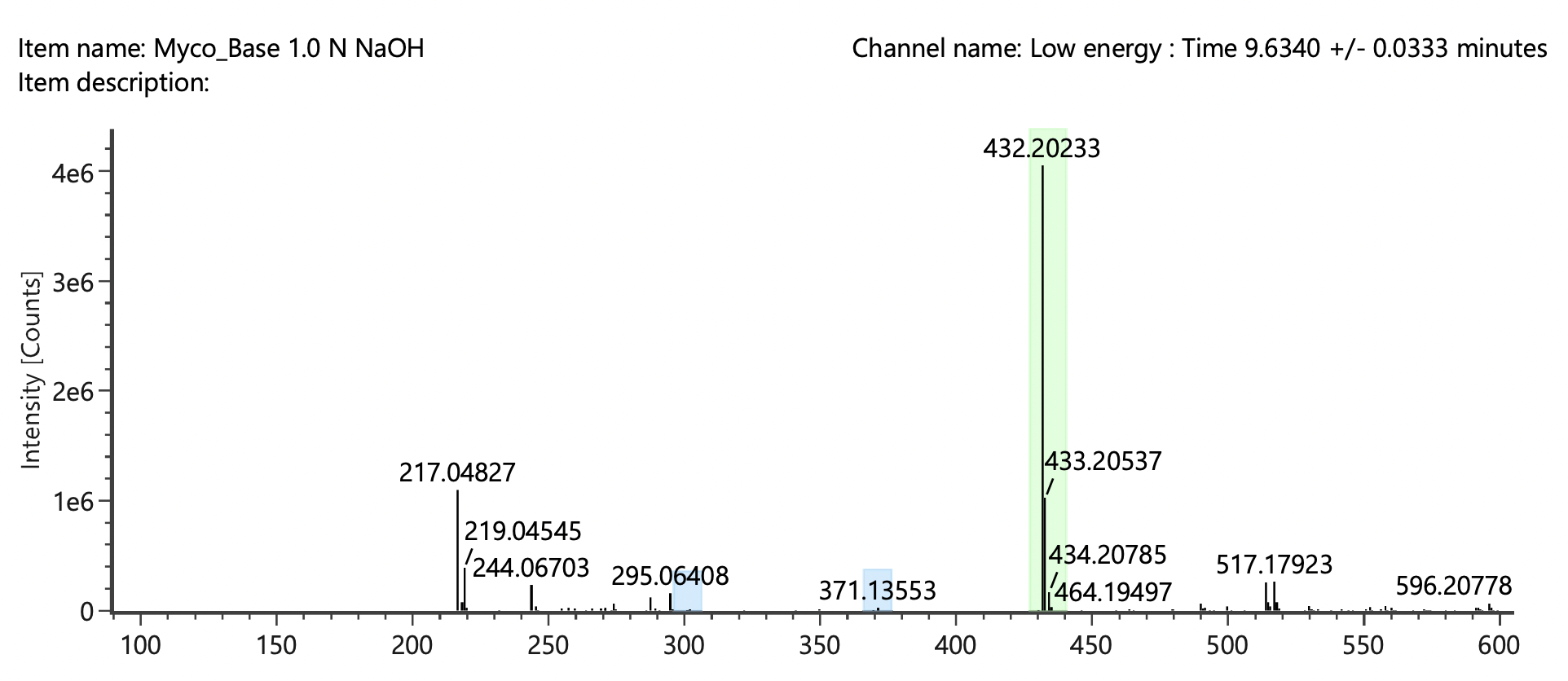


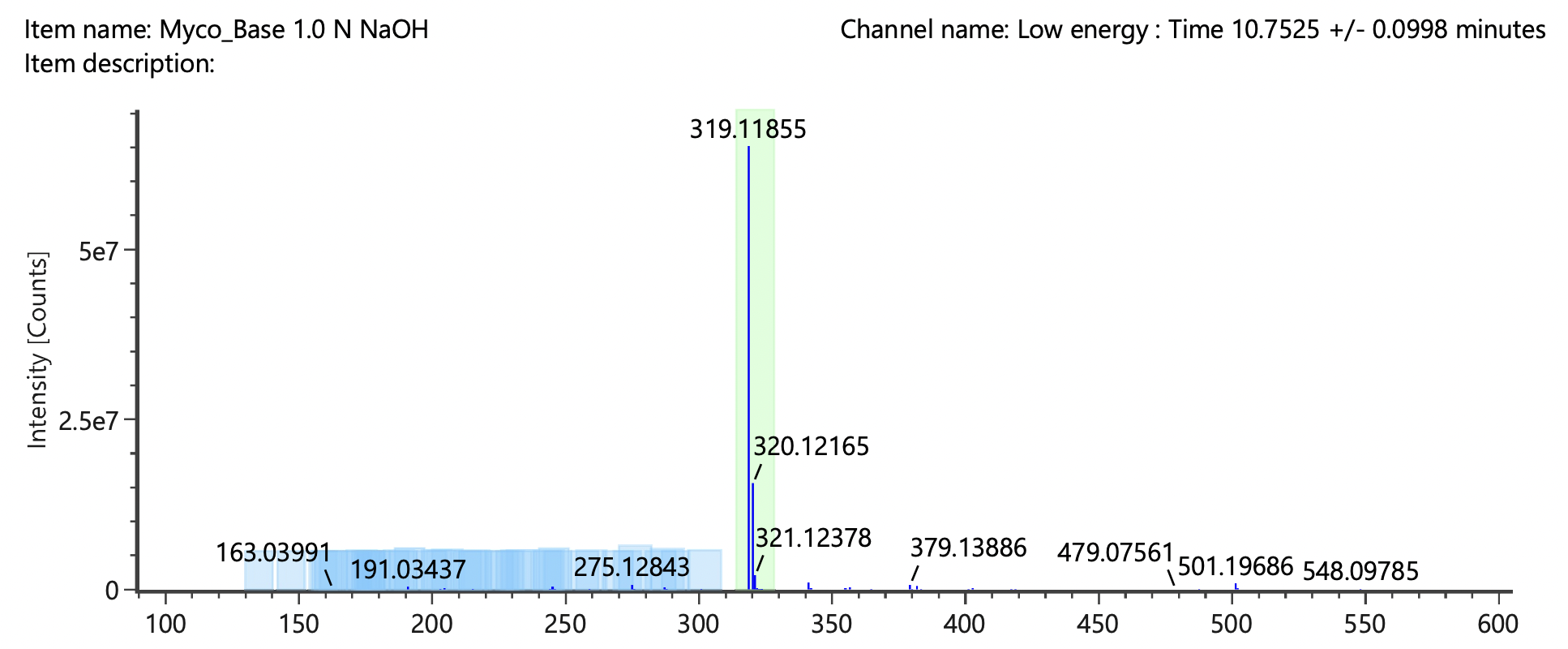


**Supplementary Fig. 10** Negative mode low energy mass spectrums. (a) Unknown at RRT 0.84; (b) Unknown at RRT 0.85;(c) Unknown at RRT 0.87; (d) Mycophenolic acid sorbitol ester, (e) MPM, and (f) MPA.


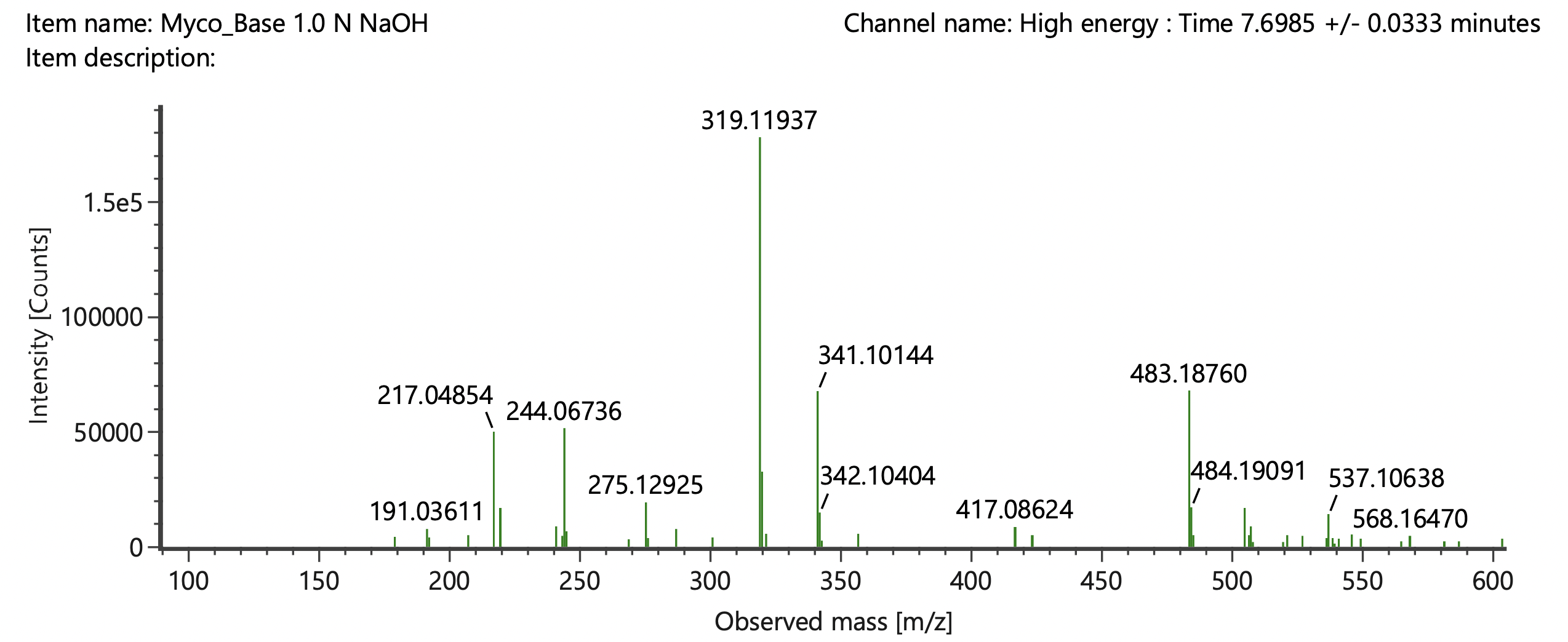


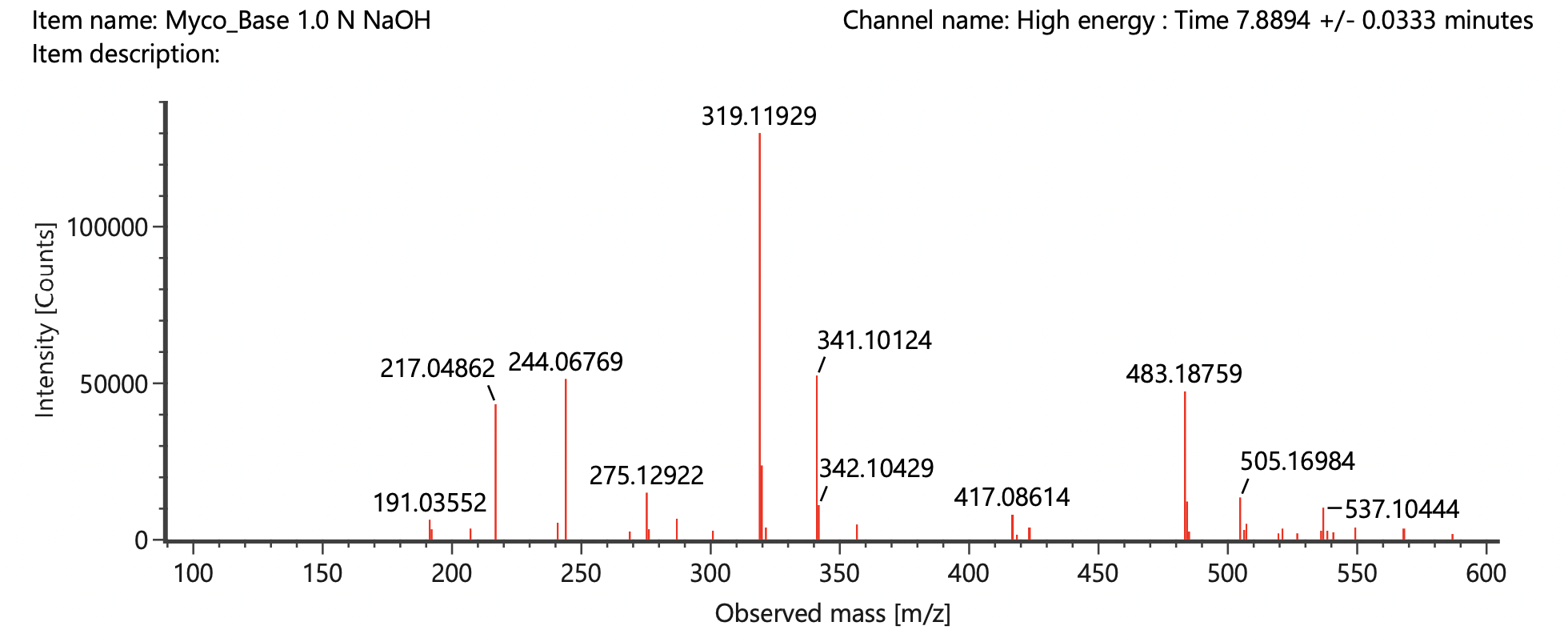


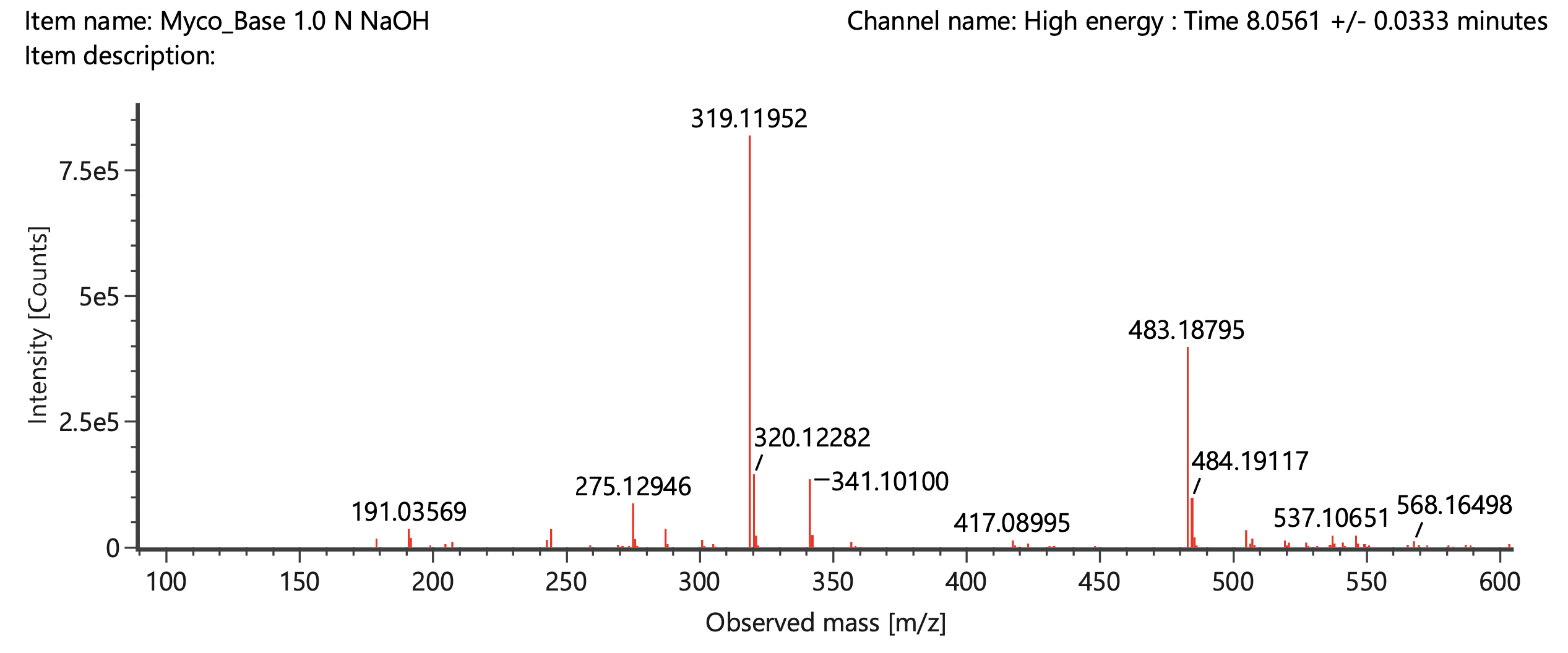


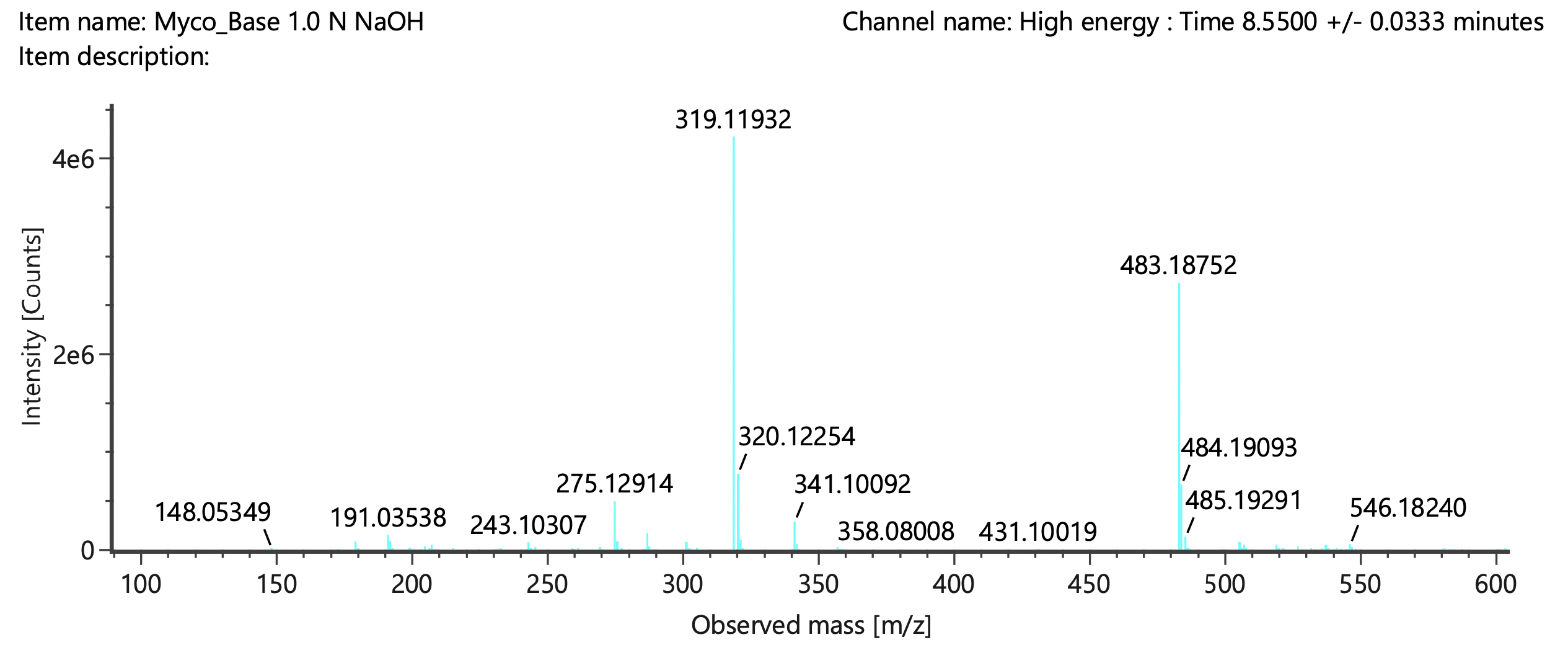


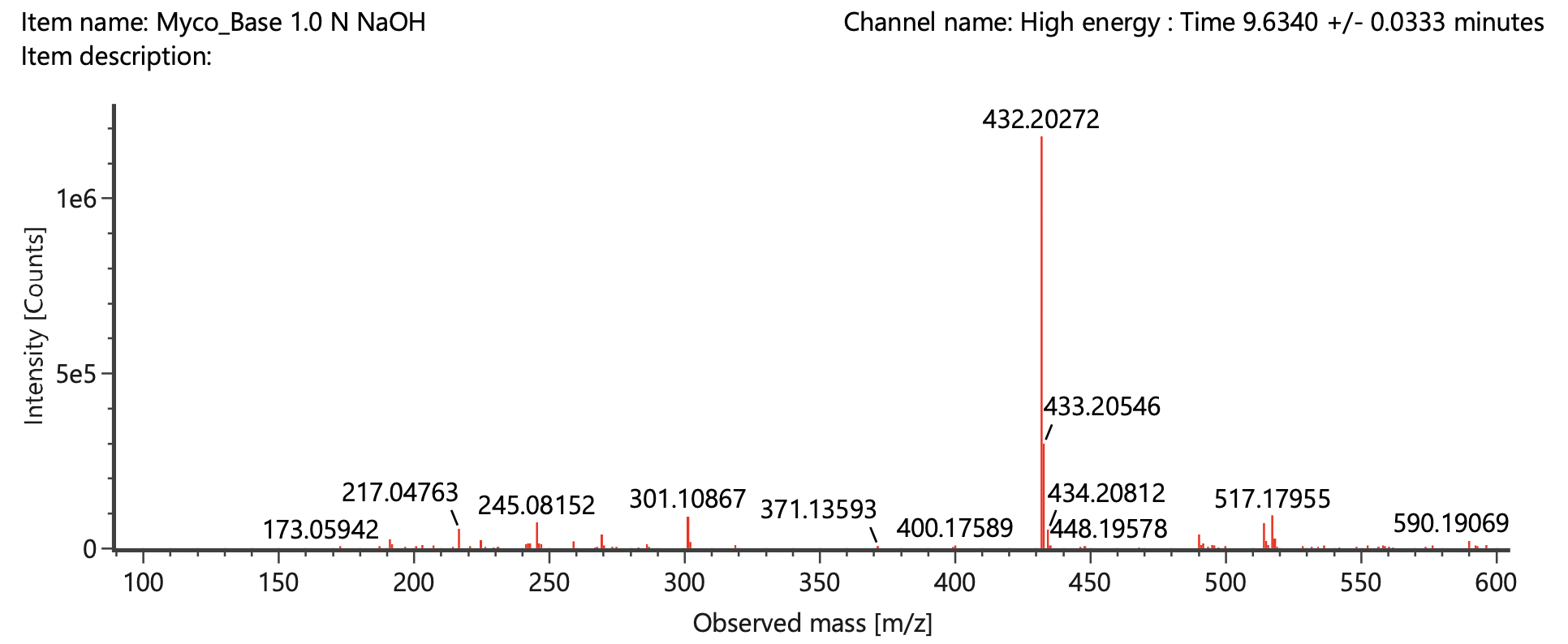


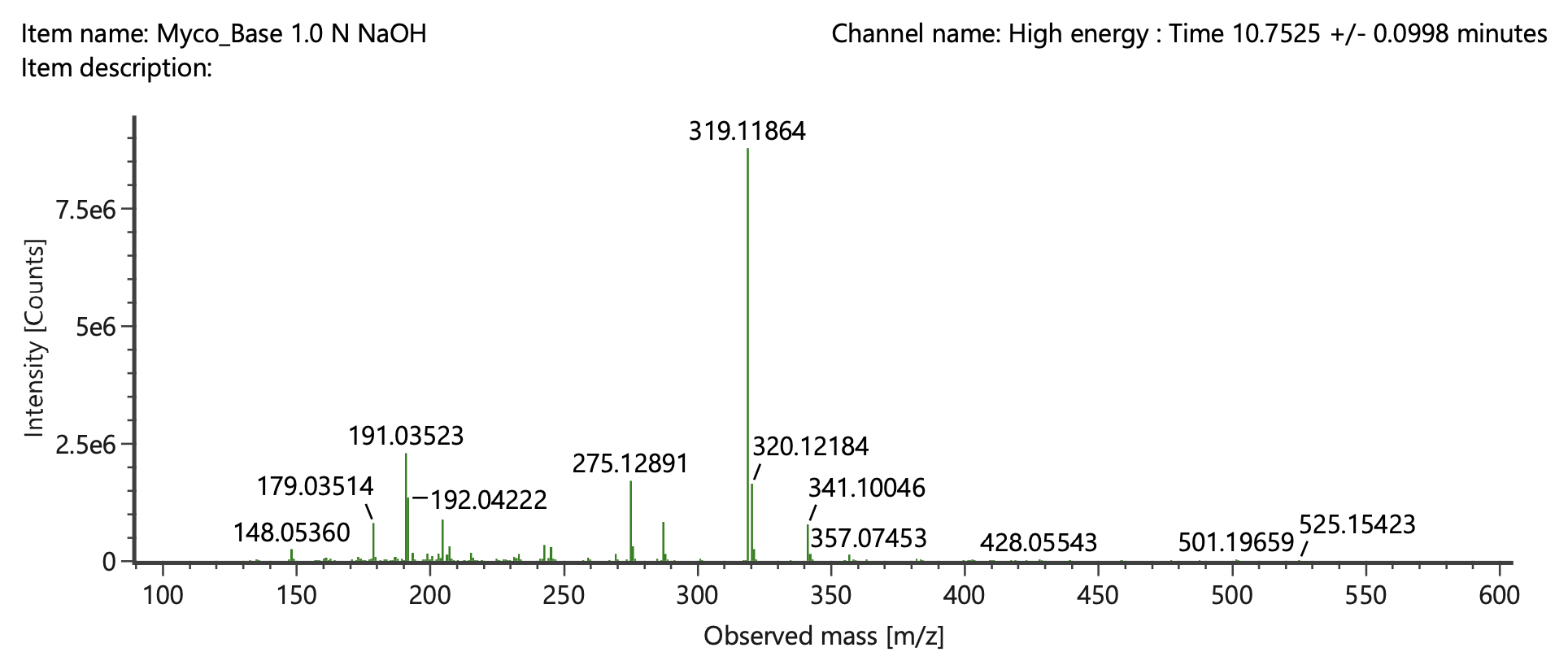


**Supplementary Fig. 11** Negative mode high energy mass spectrums. (a) Unknown at RRT 0.84; (b) Unknown at RRT 0.85;(c) Unknown at RRT 0.87; (d) Mycophenolic acid sorbitol ester, (e) MPM, and (f) MPA.
